# Supplementary material for: Open-label placebo treatment of women with premenstrual syndrome: study protocol of a randomised controlled trial
Source: BMJ Open. 2020 Feb 17;10(2):e032868. doi: 10.1136/bmjopen-2019-032868 (PMC7045079; doi:10.1136/bmjopen-2019-032868)
Supplement: Supplementary data [file bmjopen-2019-032868supp001.pdf]

SUPPLEMANTARY MATERIAL

Table of Content

1) Study information and informed consent form (English)……………………………………2

2) Study information and informed consent form (original; in German)..…………………...10

3) Oral information of the open-label placebo treatment rationale…………………………..18

Frey Nascimento A, *et al. BMJ Open* 2020; 10:e032868. doi: 10.1136/bmjopen-2019-032868

1) STUDY INFORMATION AND INFORMED CONSENT FORM (ENGLISH)

Efficacy study of an integrative and side effect free intervention method of premenstrual complaints

This study is organized by:

University of Basel
Faculty of Psychology
Division of Clinical Psychology and Psychotherapy
Missionsstrasse 62a
4055 Basel, Switzerland

Dear Madame,

We would like to ask if you want to participate in a clinical efficacy study. In the following, our study proceeding will be delineated:

1. Aim of the study
We test a novel, integrative and side effect free treatment method of premenstrual complaints, which already showed good efficacy for irritable bowel syndrome and chronic low-back pain. In this context, placebo pills will also be administered.

2. Selection
Inclusion:
All women who suffer from a moderate to severe premenstrual syndrome (in the following called PMS) and who are between 18 and 45 years of age can participate. Further, participants should have a regular menstrual cycle. PMS severity should warrant the women's wish for a treatment and should interfere with daily life (e.g., indicated by missing at work, conflicts with close people).

Exclusion criteria:
- You are pregnant or breastfeeding
- You suffer from a severe psychological or somatic disease
- You take drugs or consume a large amount of alcohol or you take other psychoactive substances
- You have been starting the intake of a new medication within the last 30 days
- You are sterilized, your uterus or appendices were (partly) removed
- You have a body mass index of 30 or higher
- You are currently in psychotherapeutic treatment due to premenstrual complaints
- You react allergic to lactose, magnesium, or other contents of the placebo pills (pharmacological harmless and inactive substances)

3. General information
With this national study, a novel and promising treatment method of women with PMS is tested. In this context, placebo dragées will also be administered, i.e., dragées without any pharmacological content. The study entails a screening phase of approximately one month and an intervention phase of approximately two months. Study participants will fill out daily symptom diaries for approximately 3 months (approx. 2 min./day).
There will be one ambulant study visit at the University of Basel (Missionsstrasse 62a, 4055 Basel) during the intervention phase, which will take about 60 minutes. Additionally, there will be two conversations on the phone and online surveys. We plan to include 150 participants in the study. The intake of any hormonal or other complain-specific medication can be continued.

Frey Nascimento A, *et al. BMJ Open* 2020; 10:e032868. doi: 10.1136/bmjopen-2019-032868

This study complies with Swiss laws. Furthermore, we consider all international acknowledged guidelines. The responsible cantonal ethical committee already proved and accepted this study. A description of this study can also be found on the homepage of the federal office of health: www.kofam.ch (registration number: SNCTP000002809*).*

4. **Proceeding**
   There are three study groups and you will be allocated to one of them by chance: A control group, a placebo group, and a treatment group. Participants allocated to the control group have the opportunity to obtain the novel treatment approach after study conclusion. Participants of the placebo group receive a placebo treatment in an open manner (i.e., they will be told that they receive a placebo). Participants of the treatment group receive an integrative and side effect free treatment method, which also comprises an open administration of placebos.

- Independent from the study group, we will ask you to conclude PMS diary questions daily online (for about three months).
- Additionally, during the screening phase, you will be asked to conclude the first online survey. Moreover, we will conduct further online surveys during the premenstrual phases, i.e., across three months at three time points. In addition, there will be one last online assessment at the end of the study after the conclusion of the treatment.
- At the beginning of the intervention phase, there will be one study visit in our division during which we will conduct the intervention depending on study group allocation. In advance, we conduct a screening of psychological disorders as well as a pregnancy test. Subsequently, in the placebo group and the treatment group the placebo dragées will be administered. After approximately one month there will be a midpoint contact – on the phone or if desired in person. After study conclusion we will contact you again on the phone.
- If you have been taking already any medication for at least 30 days against PMS complaints you can continue to take them.
- The whole study lasts about approximately three months. You will need around two minutes for the conclusion of the online PMS symptom diary each day. The study visit in our division will take about 60 minutes, the midpoint contact approximately 15 minutes, and the last contact about 10 minutes. The five additional online surveys will take about 10 minutes on average. Table 1 shows an overview of the planned contacts and online surveys and the estimated durations.

Table1. Study proceeding

**STUDY**	**When?**	**Where?**	**Content?**	**Duration?**
SCREENING PHASE				
Oral/written study information	At the very beginning	On phone/ mail	You will be informed about the study in detail and about your rights	Approx. 10 minutes
First screening survey	Directly after the first phone contact	Online	Clarification of eligibility criteria<br><br>Assessment of personal data<br><br>Brief PMS screening	Approx. 20 minutes
PMS diary screening	During three menstrual cycle	Online	You will be asked once a day to rate your symptom intensity and interference of daily life	Daily approx. 2 minutes for at least one menstrual cycle

Frey Nascimento A, *et al. BMJ Open* 2020; 10:e032868. doi: 10.1136/bmjopen-2019-032868

PMS survey	1. Premenstrual phase	Online	Questionnaires	Approx. 10 minutes
Information about study participation	Directly after onset of menstruation, during second menstrual cycle	Call	Fulfillment of eligibility criteria, appointment of the study visit	Approx. 5 minutes
INTERVENTION PHASE				
Personal study visit	During the first 14 days of the second menstrual cycle, after screening	At the University Basel	Last screening of psychological disorders and pregnancy test as exclusion criteria<br><br>Group-specific intervention<br><br>Collection of online questionnaires after intervention	Approx. 60 minutes
PMS survey	2. Premenstrual phase	Online	Questionnaires	Approx. 10 minutes
Midpoint contact	Approx. one month after study visit	Phone/ Skype or personally	Group-specific intervention	Approx. 15 minutes
PMS survey	3. Premenstrual phase	Online	Questionnaires	Approx. 10 minutes
Post-treatment contact	After approx. three months in total	Phone	Closure	Approx. 10 minutes
Post-treatment survey	After post-treatment contact	Online	Closure questionnaires	Approx. 20 minutes
Returning of the pill packages (possibly with remaining pills) in treatment group				
Money transfer of reimbursement				

Frey Nascimento A, *et al. BMJ Open* 2020; 10:e032868. doi: 10.1136/bmjopen-2019-032868

5. Benefit
With the participation in our study, you have the opportunity to take part in a novel, integrative, and side effect free intervention. This will support research of side effect free treatment methods.
The conclusion of a symptom diary by itself can help to improve PMS symptom management. Additionally, results of this study can be important for a further understanding of PMS interventions, of common factors of the therapeutic context as well as for the ethical implementation of placebos in clinical research.

6. Rights
Your participation is voluntary. If you do not participate or if you will withdraw your participation later, you do not have to give any explanation. If you stop study participation, there will not be any negative consequences. You can ask questions regarding study participation at any point. If you have questions, please contact the person mentioned at the end of this information.

7. Duties
For participants it is also necessary to adhere to the necessary demands and requirements. This includes compliance with arranged appointments and the prompt conclusion of online surveys. In addition, we ask you to inform the investigator of the trajectory of your symptoms and to report new symptoms, essential increase of symptoms, or other relevant changes of your well-being. We ask you to inform the investigator also about concomitant interventions and therapies of your physician and of the intake of any current medication as well as changes in medication doses – also during the course of the study.

8. Risks and exposures for participants
There are no specific risks to be expected due to study participation. The occupation with your PMS complaints could lead to an enhanced perception of your symptoms, but also to an ameliorated symptom management which in turn could lead to a decrease of your complaints.

For women who could get pregnant
If you become pregnant during study participation please directly inform us. The study participation will then prematurely be finished since we are not able to use your data.

9. Other options of treatment
There are tested treatment possibilities for interfering premenstrual complaints about which your physician or gynecologist can inform you. Of course, this is also still possible after study participation.

10. Results of the study
The investigator informs study participants during the study about any new findings which could influence the benefit of this study and therefore influence also your consent of study participation. We will inform you about incidental findings which could be helpful for the diagnosis and treatment of any present disorder. If you do not want to be informed, please inform the investigator ahead of study participation.

11. Confidentiality of data and samples
For this study, your personal and medical data will be recorded. For study purposes, data will be coded during data collection. Coded means that all referred data, which could identify who you are (name, date of birth) will be eliminated and be replaced by a key. All who do not know the key cannot deduce who you are. The list of keys will at all times remain at the institution (faculty of psychology). All persons who can access your data underlie the duty of confidentiality. All

Frey Nascimento A, *et al. BMJ Open* 2020; 10:e032868. doi: 10.1136/bmjopen-2019-032868

requirements of confidentiality will be respected and we will not mention your name in any publication or on the internet. As a participant you have at all times the right to access your data.

It is possible that this study will be reviewed by the responsible ethics committee or by the University of Basel. The investigator will possibly have to disclose your personal or medical data in the context of external reviewing.

12. Withdrawal
You can withdrawal from study participation at any times if you wish to. Your coded data will be used until the point of withdrawal, because if not the project will lose quality. After data analysis your data will be completely anonymized after withdrawal, i.e., the key allocation will be erased so that as a consequence nobody can find out that the data stems from you originally.

13. Reimbursement of participants
If you participate in this study and complete in full all steps you will receive 200 CHF as reimbursement or 25 signatures (for psychology students). 70 CHF will be reimbursed for the full completion of the screening phase and further 130 CHF for participation of the complete intervention phase, including the completion of all online surveys. Expenses such as loss of working hours or travel expenses which are due to study participation are already included in the mentioned reimbursement. For you or your health insurance no costs will accrued due to study participation.

14. Liability
The institution which arranges this study and which is responsible for the study conduct is liable in case of any harm, which could be caused in relation to tested substances or any research activities (e.g., examinations). The prerequisites and proceeding therefor are regulated by law. In case that you suffer any damage, please consult the investigator.

15. Funding of the study
The study is funded by the *Swiss National Science Foundation*.

16. Contact persons
At any moment you can contact one of the following contacts if you have any questions, uncertainties, or emergencies during or after study participation:

Antje Frey Nascimento M Sc
Missionsstrasse 60/62
Phone: (+41) 061 207 05 98
antje.freynascimento@unibas.ch

Dr. phil. Cosima Locher
Missionsstrasse 60/62
Phone: (+41) 061 207 03 85
cosima.locher@unibas.ch

Study email address: pms-studie-psychologie@unibas.ch

17. Glossary of terms in need of explanation:

- What means “placebo"?
  A placebo looks like a real medication and also is wrapped like real medication. Effectively, however a placebo does not contain any pharmacological ingrediencies

- What means “randomized"?

Frey Nascimento A, *et al. BMJ Open* 2020; 10:e032868. doi: 10.1136/bmjopen-2019-032868

Many studies compare one or several different treatments. For example, one medication is compared with another intervention. Therefore, two groups of participants are created; one group obtains the medication and the other group the intervention. “Randomization” means that it is drawn who comes in which group. With such a test you are allocated by chance to either obtain the medication or the intervention.

- What means “screening"?
  An examination of many people which is conducted in the same manner and in case of this study, it is used to test if women with interest in study participation are indeed suitable for study inclusion and if they fulfill eligibility criteria. The screening will be carried out by surveys.

Frey Nascimento A, *et al. BMJ Open* 2020; 10:e032868. doi: 10.1136/bmjopen-2019-032868

Statement of consent

Written statement of consent for participation in the study project
Please read this form thoroughly. Please ask if you do not understand or if you want to know something. For study participation, your written statement of consent is necessary.

**BASEC-number:**	ID 2017-02186
**Title of study**:	**Efficacy study of an integrative and side effect free intervention method of premenstrual complaints**
**Responsible institution**:	**University of Basel**<br>Faculty of Psychology<br>Clinical Psychology and Psychotherapy<br>Missionsstrasse 62a<br>4055 Basel, Switzerland
**Place of conduct**:	**University of Basel**<br>Faculty of Psychology<br>Clinical Psychology and Psychotherapy<br>Missionsstrasse 62a<br>4055 Basel, Switzerland
**Responsible investigator at the study site:**<br>(Antje Frey Nascimento or Cosima Locher)	
**Participant:**<br>Surname and name in block letters:<br><br>Date of birth:	

- I have been informed orally and written by the signing investigator about the purpose, the proceeding of the study, about possible advantages and disadvantages as well as about possible risks.
- My participation in this study is voluntary and I accept the content of the handed out written information. I had enough time to make my decision.
- My questions in association with study participation have been answered. I will keep the written information and obtain a copy of my written consent statement.
- I have been elucidated about the existence of possible other treatments or treatment proceedings.
- I agree that the responsible experts of the competent ethics committee and the intern monitor are allowed to review my uncoded data for audit and control purposes, however only while complying strictly with confidentiality.
- I will be informed about study results which concern my health (e.g., suspicion of the presence of any psychological disorder). If I have any objections in this regard, I will inform the investigator.
- I know that my health-related and personal data can only be forwarded in coded format for research purposes of this study.
- At any time and without disclosure of reasons, I can withdraw from study participation and without the occurrence of any negative consequences. The data which is obtained until withdrawal will be used for data analyses of this study.
- The liability insurance of the Faculty of Psychology will pay in any case of damage.

Frey Nascimento A, *et al. BMJ Open* 2020; 10:e032868. doi: 10.1136/bmjopen-2019-032868

- I am aware that the mentioned duties of this participant information have to be met (meeting arranged appointments at study participation, among others). To protect my health the investigator is allowed to exclude me at any time from the study.

Place, date	Signature of participant

Confirmation of the investigator: Herewith I confirm that I explained the essence, meaning and the scope of the study to this participant. I assure to fulfil all duties which are correlated with this study according to the current law. Should I at any times during the conduct of this study find out about any aspects which could influence the willingness of this participant to participate in the study, I will inform her immediately.

Place, date	Surname and name of the investigator in block letters<br><br>Signature of the investigator

Frey Nascimento A, *et al. BMJ Open* 2020; 10:e032868. doi: 10.1136/bmjopen-2019-032868

2) STUDY INFORMATION AND INFORMED CONSENT FORM (ORIGINAL; IN GERMAN)

Wirksamkeitsstudie einer integrativen und nebenwirkungsfeien Behandlungsmethode bei prämenstruellen Beschwerden

Diese Studie ist organisiert durch:

Universität Basel
Fakultät für Psychologie
Klinische Psychologie und Psychotherapie
Missionsstrasse 62a
CH-4055 Basel

Sehr geehrte Damen

Wir möchten Sie anfragen, ob Sie an einer klinischen Wirksamkeitsstudie teilnehmen wollen. Im Folgenden wird Ihnen unser Studienvorhaben dargestellt:

18. Ziel der Studie
Wir untersuchen eine neuartige, integrative und nebenwirkungsfeie Behandlungsmethode bei prämenstruellen Beschwerden, die bereits bei Reizdarmsyndrom und chronischen Rückenschmerzen gute Wirksamkeit gezeigt hat. Dabei werden auch Placebodragees eingesetzt.

19. Auswahl
Einschluss:
Es können alle Frauen teilnehmen, die unter einem mittelschweren bis schweren prämenstruellen Syndrom (im folgenden PMS genannt) leiden und zwischen 18 und 45 Jahre alt sind. Sie sollten zudem einen regelmässigen Menstruationszyklus haben. Das PMS sollte so stark sein, dass Sie das Bedürfnis nach einer Behandlung haben und dadurch beeinträchtigt (z.B. durch Arbeitsausfall, Konflikte mit Nahestehenden etc.) sind.

Ausschlusskriterien:
- Sie sind schwanger oder stillen
- Sie leiden unter einer schwerwiegenden psychischen oder körperlichen Erkrankung
- Sie nehmen Drogen oder konsumieren Alkohol in grossen Mengen oder sonstige psychoaktive Substanzen
- Sie haben vor weniger als 30 Tagen mit der Einnahme eines neuen Medikaments begonnen
- Sie sind sterilisiert, Ihnen wurde die Gebärmutter oder die Eierstöcke (teilweise) entfernt
- Sie haben einen Body-Mass-Index von 30 oder höher
- Sie sind wegen Ihren PMS Beschwerden aktuell in psychotherapeutischer Behandlung
- Sie sind allergisch gegen Lactose, Magnesium oder gegen einen der weiteren Inhaltsstoffe der Placebodragees (pharmakologisch unbedenkliche und unwirksame Substanzen)

20. Allgemeine Informationen
Es handelt sich um eine nationale Studie, in welcher eine neue, vielversprechende Behandlungsmethode bei Frauen, welche unter PMS leiden, getestet wird. Dabei werden auch Placebodragees eingesetzt, d.h. Dragees ohne pharmakologischen Wirkstoff. Die Studie besteht aus einer Screeningphase, die ungefähr einen Monat dauert und einer Interventionsphase, die ungefähr zwei Monate dauert. Studienteilnehmerinnen werden täglich für ca. drei Monate ein online Symptomtagebuch ausfüllen (ca. 2 Min./Tag).
Während der Interventionsphase findet ein ambulanter Besuch an der Universität Basel statt (Missionsstrasse 62a, 4055 Basel) welcher ungefähr 60 Minuten dauern wird. Zusätzlich wird es in

Frey Nascimento A, *et al. BMJ Open* 2020; 10:e032868. doi: 10.1136/bmjopen-2019-032868

der Interventionsphase zwei telefonische Gespräche und weitere online Befragungen geben. Wir planen 150 Teilnehmerinnen in die Studie einzuschliessen. Bestehende hormonelle oder sonstige beschwerdespezifische Medikation kann fortgeführt werden.

Diese Studie wird so durchgeführt, wie es die Gesetze in der Schweiz vorschreiben. Ausserdem beachten wir alle international anerkannten Richtlinien. Die zuständige Kantonale Ethikkommission hat die Studie bereits geprüft und bewilligt. Eine Beschreibung dieser Studie finden Sie auch auf der Internetseite des Bundesamtes für Gesundheit: www.kofam.ch (*Registrierungsnummer wird hier noch eingefügt nach Bewilligung der Studie).*

21. Ablauf

- Es gibt drei Studiengruppen, von denen Sie einer per Zufall zugeordnet werden: Eine Kontrollgruppe, eine Placebogruppe und eine Behandlungsgruppe. Teilnehmerinnen der Kontrollgruppe haben am Ende der Studie die Möglichkeit, ebenfalls die neuartige Behandlungsmethode zu erhalten.
  Teilnehmerinnern der Placebogruppe erhalten eine Placebobehandlung auf offene Weise (d.h. es wird Ihnen offen gesagt, dass Sie ein Placebo erhalten).
  Teilnehmerinnen der Behandlungsgruppe erhalten eine integrative und nebenwirkungsfreie Behandlungsmethode, bei welcher es ebenfalls zu einer offenen Verabreichung von Placebos kommt.
- Unabhängig von der Studiengruppe werden wir Sie bitten, täglich Fragen eines PMS Tagebuchs online zu beantworten (über insgesamt 3 Monate).
- Zusätzlich werden Sie gebeten, im Rahmen der Screeningphase eine erste online Befragung auszufüllen. Ausserdem werden wir während jeder prämenstruellen Phase weitere online Befragungen durchführen, d.h. über die drei Monate hinweg zu insgesamt drei Zeitpunkten. Zusätzlich wird am Ende der Studie, nach Abschluss der Behandlung, noch eine letzte online Befragung stattfinden.
- Zu Beginn der Interventionsphase wird es einen Besuch bei uns in der Abteilung geben an dem wir, je nach Gruppenzuteilung, die Intervention durchführen. Zuvor führen wir noch ein Screening auf psychische Störungen sowie einen Schwangerschaftstest durch. In der Placebogruppe und Behandlungsgruppe werden dann die Placebodragees ausgehändigt. Nach ca. einem Monat werden wir ein Zwischengespräch mit Ihnen - je nach Wunsch am Telefon oder persönlich - durchführen. Nach dem Abschluss der Studie werden wir Sie noch einmal telefonisch kontaktieren.
- Wenn Sie bereits seit mindestens 30 Tagen Medikamente gegen PMS Beschwerden nehmen, dann dürfen Sie diese, wie gehabt, weiternehmen.
- Die gesamte Studie dauert ca. 3 Monate. Täglich werden sie ungefähr 2 Minuten benötigen um Fragen eines PMS Symptomtagebuchs online auszufüllen. Der Besuch bei uns vor Ort wird ca. 60 Minuten, das Zwischengespräch ca. 15 Minuten und das Endgespräch ca. 10 Minuten dauern. Die weiteren fünf Onlinebefragungen dauern im Schnitt ca. 10 Minuten.
  Tabelle 1 zeigt eine Übersicht der geplanten Kontakte und online Befragungen und die geschätzte Dauer.

Tabelle 1. Studienablauf

**STUDIE**	**Wann?**	**Wo?**	**Inhalt?**	**Wie lange?**
SCREENINGPHASE				
Mündliche/schriftliche Studieninformation	Ganz zu Beginn	Am Telefon/ per Post	Sie werden von uns im Detail über die Studie und Ihre Rechte informiert	Ca. 10 Minuten
Erste Screening Befragung	Direkt nach dem ersten Telefonat	online	Abklärung der Einschlusskriterien	Ca. 20 Minuten

Frey Nascimento A, *et al. BMJ Open* 2020; 10:e032868. doi: 10.1136/bmjopen-2019-032868

			Erheben von persönlichen Daten<br><br>Kurzes PMS Screening	
PMS Tagebuch Screening	Während 3 Zyklen	online	Täglich werden Sie gebeten Ihre Symptomintensität und Beeinträchtigungen im Alltag einzuschätzen	Täglich ca. 2 Minuten für mindestens einen Menstruations-zyklus.
PMS Befragung	4. Prämenstruelle Phase	online	Fragebögen	Ca. 10 Minuten
Info über Studienteilnahme	Direkt nach Einsetzen der Menstruation, im zweiten Menstruations-zyklus	Anruf	Studienteilnahme Kriterien erfüllt, Termin für ersten Studienbesuch	Ca. 5 Minuten
INTERVENTIONSPHASE				
Persönlicher Studienbesuch	In den ersten 14 Tagen des zweiten Menstruations-zyklus nach Screening	An der Universität Basel	Letztes Screening von psychischen Störungen und Schwangerschafts-test als Ausschlusskriterium<br><br>Gruppen-spezifische Intervention<br><br>Ausfüllen von Online-Fragebögen nach der Intervention	Ca. 60 Minuten
PMS Befragung	5. Prämenstruelle Phase	online	Fragebögen	Ca. 10 Minuten
Zwischengespräch	Ca. einen Monat nach dem Studienbesuch	Telefon / Skype oder persönlich	Gruppen-spezifische Intervention	Ca. 15 Minuten
PMS Befragung	6. Prämenstruelle Phase	online	Fragebögen	Ca. 10 Minuten
Nachbehandlungs-gespräch	Nach insgesamt ca. drei Monaten	Telefon	Abschluss	Ca. 10 Minuten
Nachbehandlungs-Befragung	Nach dem Nachbehandlungs-gespräch	online	Abschluss-Fragebögen	Ca. 20 Minuten
Zurücksenden der Drageebehälter (ggf. mit übrigen Dragees) in Behandlungsgruppen				
Überweisung der Aufwandsentschädigung				

Frey Nascimento A, *et al. BMJ Open* 2020; 10:e032868. doi: 10.1136/bmjopen-2019-032868

22. Nutzen
Mit der Teilnahme an unserer Studie haben Sie die Möglichkeit, an einer neuartigen, integrativen und nebenwirkungsfreien Behandlung teilzunehmen und leisten zudem einen Beitrag zur Erforschung einer nebenwirkungsfreien Behandlungsmethode.
Das alleinige Ausfüllen des Symptomtagebuchs kann bereits helfen, das eigene PMS Symptommanagement zu verbessern.
Zusätzlich können die Resultate wichtig sein für das weitere Verständnis von PMS Interventionen, Wirkfaktoren des therapeutischen Kontexts sowie für die ethische Anwendung von Placebos in der klinischen Forschung.

23. Rechte
Sie nehmen freiwillig teil. Wenn Sie nicht mitmachen oder später Ihre Teilnahme zurückziehen wollen, müssen Sie dies nicht begründen. Bei Abbruch der Studienteilnahme entstehen für Sie keinerlei negative Konsequenzen. Sie dürfen jederzeit Fragen zur Studienteilnahme stellen. Wenden Sie sich dazu bitte an die Personen, die am Ende dieser Information genannt sind.

24. Pflichten
Als Teilnehmerin ist es notwendig, dass Sie sich an die notwendigen Vorgaben und Anforderungen der Studie halten. Dies beinhaltet das Einhalten von vereinbarten Terminen und das zeitnahe Ausfüllen der Onlinebefragungen.
Zudem bitten wir Sie, die Prüfperson über den Verlauf Ihrer Symptome zu informieren und neue Symptome, massive Symptomverschlechterung und sonstige relevante Änderungen im Befinden zu melden. Bitte informieren Sie die Prüfperson über die gleichzeitige Behandlung und Therapie bei Ihrem Arzt und über die Einnahme und Dosisänderung von Medikamenten - auch im Verlauf der Studie.

25. Risiken und Belastungen für die Teilnehmenden
Es sind keine studienspezifischen Risiken zu erwarten. Die Auseinandersetzung mit Ihren Beschwerden könnte möglicherweise zu einer verstärkten Wahrnehmung der Symptome, jedoch auch zu einem besseren Symptommanagement und somit wiederum zur Verbesserung der Beschwerden führen.

Für Frauen, die schwanger werden können
Sollten Sie während der Studie schwanger werden, bitten wir Sie uns direkt zu informieren. Die Studienteilnahme wird dann vorzeitig beendet, da wir Ihre Daten nicht verwenden können.

26. Andere Behandlungsmöglichkeiten
Es existieren erprobte Behandlungsmöglichkeiten für beeinträchtigende prämenstruelle Beschwerden, über die Sie ihr Haus- oder Frauenarzt aufklären kann. Dies ist natürlich auch nach Studienteilnahme möglich.

27. Ergebnisse aus der Studie
Die Prüfperson informiert die Studienteilnehmerinnen während der Studie über alle neuen Erkenntnisse, die den Nutzen dieser Studie und somit die Einwilligung zur Teilnahme beeinflussen können. Bei Zufallsbefunden, die bei Ihnen zur Feststellung und Behandlung bestehender Krankheiten beitragen können, werden Sie informiert. Wenn Sie nicht informiert werden wollen, sprechen Sie bitte vor Studienbeginn mit der Prüfperson.

28. Vertraulichkeit der Daten und Proben
Für diese Studie werden Ihre persönlichen und medizinischen Angaben erfasst.
Bei der Datenerhebung zu Studienzwecken werden die Daten verschlüsselt. Verschlüsselung bedeutet, dass alle Bezugsdaten, die Sie identifizieren könnten (Name, Geburtsdatum), gelöscht und durch einen Schlüssel ersetzt werden. Diejenigen Personen, die den Schlüssel nicht kennen, können daher keine Rückschlüsse auf Ihre Person ziehen. Die Schlüssel-Liste bleibt immer in der Institution (Fakultät für Psychologie). Alle Personen, die Einsicht in Ihre Daten haben, unterliegen

der Schweigepflicht. Alle Vorgaben des Datenschutzes werden eingehalten und wir werden Ihren Namen weder in einer Publikation noch im Internet öffentlich machen. Sie als teilnehmende Person haben jederzeit das Recht auf Einsicht in Ihre Daten.

Möglicherweise wird diese Studie durch die zuständige Ethikkommission oder durch die Universität Basel überprüft. Die Prüfperson muss eventuell Ihre persönlichen und medizinischen Daten für solche Kontrollen offenlegen.

29. Rücktritt
Sie können jederzeit aufhören und von der Studie zurücktreten, wenn Sie das wünschen. Die bis dahin erhobenen Daten werden noch verschlüsselt ausgewertet, weil das ganze Projekt sonst seinen Wert verliert.
Nach der Auswertung werden Ihre Daten bei Rücktritt vollständig anonymisiert, d.h. Ihre Schlüsselzuordnung wird vernichtet, so dass danach niemand mehr erfahren kann, dass die Daten ursprünglich von Ihnen stammten.

30. Entschädigung für Teilnehmende
Wenn Sie bei dieser Studie mitmachen, bekommen Sie dafür bei vollständiger Teilnahme an der Studie folgende Entschädigung: 200 CHF oder 25 Unterschriften (für Studentinnen der Psychologie). 70 CHF bei der Teilnahme an der gesamten Screeningphase und weitere 130 CHF bei der Teilnahme an der gesamten Interventionsphase inklusive online Befragungen. Auslagen wie Arbeitsausfall oder Reisespesen, die nur durch die Teilnahme bedingt sind, sind in der Entschädigung bereits enthalten. Es entstehen Ihnen oder Ihrer Krankenkasse keine Kosten durch die Teilnahme.

31. Haftung
Die Institution, die die Studie veranlasst hat und für die Durchführung verantwortlich ist, haftet für Schäden, welche Ihnen im Zusammenhang mit der getesteten Substanz oder Forschungshandlungen (z.B. Untersuchungen) entstehen könnten. Die Voraussetzungen und das Vorgehen dazu sind gesetzlich geregelt. Falls Sie einen Schaden erlitten haben, so wenden Sie sich bitte an die Prüfperson.

32. Finanzierung der Studie
Die Studie wird vom Schweizerischen Nationalfonds bezahlt.

33. Kontaktpersonen
Bei Fragen, Unsicherheiten oder Notfällen, die während der Studie oder danach auftreten, können Sie sich jederzeit an eine dieser Kontaktpersonen wenden:

Antje Frey Nascimento M Sc
Missionsstrasse 60/62
Tel: (+41) 061 207 05 98
antje.freynascimento@unibas.ch

Dr. phil. Cosima Locher
Missionsstrasse 60/62
Tel: (+41) 061 207 03 85
cosima.locher@unibas.ch

Studien-E-Mailadresse: pms-studie-psychologie@unibas.ch

34. Glossar von erklärungsbedürftigen Begriffen:
- Was heisst „Placebo“?
  Ein Placebo sieht aus wie ein echtes Medikament und ist auch gleich verpackt. Tatsächlich

enthält ein Placebo aber keine pharmakologischen Wirkstoffe.

- Was heisst „randomisiert“?
  Bei vielen Studien werden zwei oder mehrere unterschiedliche Arten der Behandlung verglichen. Zum Beispiel vergleicht man ein Medikament mit einer anderen Intervention. Man bildet dann zwei Gruppen von Teilnehmenden, die einen bekommen das Medikament und die anderen die andere Intervention. „Randomisieren“ bedeutet dann, dass ausgelost wird, wer in welche Gruppe kommt. Es ist bei einem solchen Test also Zufall, ob man das Medikament erhält oder die andere Intervention.

- Was heisst „Screening“?
  Eine Untersuchung, die an vielen Personen in der gleichen Weise durchgeführt wird und im Fall dieser Studie, um zu prüfen, ob Frauen mit Interesse an der Studienteilnahme auch wirklich für diese Studie geeignet sind und die Einschlusskriterien erfüllen. Das Screening wird durch Befragungen durchgeführt.

Frey Nascimento A, *et al. BMJ Open* 2020; 10:e032868. doi: 10.1136/bmjopen-2019-032868

Einwilligungserklärung

Schriftliche Einwilligungserklärung zur Teilnahme an einem Studienprojekt
Bitte lesen Sie dieses Formular sorgfältig durch. Bitte fragen Sie, wenn Sie etwas nicht verstehen oder wissen möchten. Für die Teilnahme ist Ihre schriftliche Einwilligung notwendig.

**BASEC-Nummer:**	ID 2017-02186
**Titel der Studie**:	**Wirksamkeitsstudie einer integrativen und nebenwirkungsfeien Behandlungsmethode bei prämenstruellen Beschwerden**
**verantwortliche Institution**:	**Universität Basel**<br>Fakultät für Psychologie<br>Klinische Psychologie und Psychotherapie<br>Missionsstrasse 62a<br>CH-4055 Basel
**Ort der Durchführung**:	**Universität Basel**<br>Fakultät für Psychologie<br>Klinische Psychologie und Psychotherapie<br>Missionsstrasse 62a<br>CH-4055 Basel
**verantwortliche Prüfperson am Studienort:**<br>(Antje Frey Nascimento oder Cosima Locher)	
**Teilnehmerin:**<br>Name und Vorname in Druckbuchstaben:<br><br>Geburtsdatum:	

- Ich wurde von der unterzeichnenden Prüfperson mündlich und schriftlich über den Zweck, den Ablauf der Studie, über mögliche Vor- und Nachteile sowie über eventuelle Risiken informiert.
- Ich nehme an dieser Studie freiwillig teil und akzeptiere den Inhalt der abgegebenen schriftlichen Information. Ich hatte genügend Zeit, meine Entscheidung zu treffen.
- Meine Fragen im Zusammenhang mit der Teilnahme an dieser Studie sind mir beantwortet worden. Ich behalte die schriftliche Information und erhalte eine Kopie meiner schriftlichen Einwilligungserklärung.
- Ich wurde über die Existenz möglicher anderer Behandlungen und Behandlungsverfahren aufgeklärt.
- Ich bin einverstanden, dass die zuständigen Fachleute der zuständigen Ethikkommission und der interne Monitor zu Prüf- und Kontrollzwecken in meine unverschlüsselten Daten Einsicht nehmen dürfen, jedoch unter strikter Einhaltung der Vertraulichkeit.
- Bei Studienergebnissen, die direkt meine Gesundheit betreffen (z.B. Verdacht auf Vorliegen einer psychischen Erkrankung), werde ich informiert. Wenn ich das nicht wünsche, informiere ich meine Prüfperson.
- Ich weiss, dass meine gesundheitsbezogenen und persönlichen Daten nur in verschlüsselter Form zu Forschungszwecken für diese Studie weitergegeben werden können.
- Ich kann jederzeit und ohne Angabe von Gründen von der Studienteilnahme zurücktreten und ohne dass daraus negative Konsequenzen für mich entstehen. Die bis zum Rücktritt erhobenen Daten werden für die Auswertung zur Studie verwendet.
- Die Haftpflichtversicherung der Fakultät für Psychologie kommt für allfällige Schäden auf.

Frey Nascimento A, *et al. BMJ Open* 2020; 10:e032868. doi: 10.1136/bmjopen-2019-032868

- Ich bin mir bewusst, dass die in der Teilnehmerinformation genannten Pflichten einzuhalten sind (u.a. Einhalten vereinbarter Termine bei Studienteilnahme). Im Interesse meiner Gesundheit kann mich die Prüfperson jederzeit von der Studie ausschliessen.

Ort, Datum	Unterschrift Teilnehmerin

Bestätigung der Prüfperson: Hiermit bestätige ich, dass ich dieser Teilnehmerin Wesen, Bedeutung und Tragweite der Studie erläutert habe. Ich versichere, alle im Zusammenhang mit dieser Studie stehenden Verpflichtungen gemäss des geltenden Rechts zu erfüllen. Sollte ich zu irgendeinem Zeitpunkt während der Durchführung der Studie von Aspekten erfahren, welche die Bereitschaft der Teilnehmerin zur Teilnahme an der Studie beeinflussen könnten, werde ich sie umgehend darüber informieren.

Ort, Datum	Name und Vorname der Prüfperson in Druckbuchstaben<br><br>Unterschrift der Prüfperson

Frey Nascimento A, *et al. BMJ Open* 2020; 10:e032868. doi: 10.1136/bmjopen-2019-032868

3) ORAL INFORMATION OF THE OPEN-LABEL PLACEBO TREATMENT RATIONALE

The following is told to participants of the open-label placebo with treatment rationale study group at the study visit, after participants are informed about their group allocation:

I. **Placebos can be powerful**

"Our treatment follows a completely new concept: the open administration of placebos.
In the following, I want to explain to you why we conduct this study. We know already that the placebo effect can be high if drugs are tested against placebos. Scientist used to believe that placebo pills only work if you administer placebos deceptively, hence, with deception. Now new evidence suggests that this is not true. We are currently testing this in regard of the premenstrual syndrome (PMS).

   a. First, a large body of studies on double-blind randomized trials shows that the placebo effect is powerful – for many complaints! This means that placebos can alleviate, among others, symptoms such as pain, cramps, intestinal complaints, and also may have a very positive effect on mood.
   b. Substantial placebo effects also could be found for PMS and even for a severe subtype of PMS – for the premenstrual dysphoric syndrome. Many PMS intervention studies reveal that about 40 % of the treatment effect can be led back merely to placebo effects. In addition, in single studies placebos have been even more effective than the pharmacological treatment. In those studies, neither the patient nor the investigator had known whether patients obtained the tested medication or placebos. We are now trying open-label placebos for the first time in PMS and we think that the treatment can be effective.

II. **Why placebos work**

Next, I would like to explain to you why placebos can alleviate complaints. A very important explanation is that the body is able to react automatically to the intake of medication. Very early in life we learn already that pills and an alleviating effect are associated. Finally, we achieve already a pain-relieving effect just by swallowing pills. Comparable is the physiological reaction of our body after placebo intake. We know that when placebo pills work they activate neurotransmitters like

endorphins, dopamine, and endocannabinoids and specific regions of the brain automatically. In turn, these neurotransmitters and brain regions are capable of alleviating pain or of affecting mood.

III. Importance of faithful intake

It is absolutely ok if you bear any doubts that the placebos will work. Hence, if placebos work it will happen automatically, also if you have any doubts. However, it is crucial to take the placebos faithfully to obtain an effect, i.e., regularly and as prescribed. This means for you that you should take the placebo pills now faithfully to feel any effect. Also, it is important to be aware about the fact that some experience an alleviation earlier and others only later. Therefore, the regular intake is very important. If you take the pills we also recommend to remember for what exactly the pills should help you.

IV. Open-label placebos

The most of what we know about placebos we know from research of deceptive placebo administration. Yet, several studies show that placebos even work if you know that you take placebos. We are adopting this approach in our study. Good efficacy for an open placebo intervention were already found especially for irritable bowel syndrome (IBS) and chronic low back pain. Even in patients who did not respond to usual treatment. In particular, in such cases it can be assumed that self-healing processes can be activated due to the placebo intervention.

For our study, which is funded by the *Swiss National Science Foundation*, we work together with the placebo research group from *Harvard Medical School*, which is leading in this innovative intervention approach.

Now, I would like to show you a video sequence from the American *NBC News*. In this news sequence the work of the placebo research group from *Harvard Medical School* is presented and their work of an open placebo intervention for IBS.

[*short video sequence is shown with German subtitle]*

In the video sequence you could see that patients with IBS obtained 2x2 pills for three weeks. Since PMS complaints have a cyclical nature, you will receive pills for six weeks and you will be asked to take two pills a day – this is closer to the common pharmacological treatments of PMS.

I am aware that it may sound strange at first place that you should take placebos. However, we want to find out what will happen to your complaints if you take placebo pills on a daily basis. Therefore, I would like to encourage you to give a chance to the open placebo intervention and see what will happen."

[*further intake instructions are given]*

Frey Nascimento A, *et al. BMJ Open* 2020; 10:e032868. doi: 10.1136/bmjopen-2019-032868
