# Supplementary material for: Open-label placebo treatment of women with premenstrual syndrome: study protocol of a randomised controlled trial
Source: BMJ Open. 2020 Feb 17;10(2):e032868. doi: 10.1136/bmjopen-2019-032868 (PMC7045079; doi:10.1136/bmjopen-2019-032868)
Supplement: Author's manuscript [file bmjopen-2019-032868.draft_revisions.pdf]

BMJ Open is committed to open peer review. As part of this commitment we make the peer review history of every article we publish publicly available.

When an article is published we post the peer reviewers' comments and the authors' responses online. We also post the versions of the paper that were used during peer review. These are the versions that the peer review comments apply to.

The versions of the paper that follow are the versions that were submitted during the peer review process. They are not the versions of record or the final published versions. They should not be cited or distributed as the published version of this manuscript.

BMJ Open is an open access journal and the full, final, typeset and author-corrected version of record of the manuscript is available on our site with no access controls, subscription charges or pay-per-view fees (http://bmjopen.bmj.com).

If you have any questions on BMJ Open's open peer review process please email info.bmjopen@bmj.com

BMJ Open

OPEN-LABEL PLACEBO TREATMENT OF WOMEN WITH PREMENSTRUAL SYNDROME: STUDY PROTOCOL OF A RANDOMIZED CONTROLLED TRIAL

Journal:	*BMJ Open*
Manuscript ID	bmjopen-2019-032868
Article Type:	Protocol
Date Submitted by the Author:	09-Jul-2019

Complete List of Authors:	Frey Nascimento, Antje; University of Basel<br>Gaab, Jens; University of Basel<br>Kirsch, Irving; Harvard Medical School<br>Kossowsky, Joe; University of Basel; Harvard Medical School<br>Meyer, Andrea; University of Basel<br>Locher, Cosima; University of Basel; University of Plymouth

Keywords:	open-label placebos, premenstrual syndrome, randomized controlled trial, treatment rationale, PMS symptom diary

SCHOLARONE™ Manuscripts

STUDY PROTOCOL

OPEN-LABEL PLACEBO TREATMENT OF WOMEN WITH PREMENSTRUAL SYNDROME: STUDY PROTOCOL OF A RANDOMIZED CONTROLLED TRIAL

Antje Frey Nascimento[1], Jens Gaab[1], Irving Kirsch[2], Joe Kossowsky[1, 2, 3], Andrea Meyer[4], Cosima Locher[1, 5]

[1] Division of Clinical Psychology and Psychotherapy, Department of Psychology, University of Basel, Switzerland

[2] Program in Placebo Studies, Beth Israel Deaconess Medical Center, Harvard Medical School, Boston, MA, USA

[3] Department of Anesthesiology, Critical Care & Pain Medicine, Boston Children's Hospital, Harvard Medical School, Boston, MA, USA

[4] Division of Clinical Psychology and Epidemiology, Department of Psychology, University of Basel, Switzerland

[5] University of Plymouth; School of Psychology, UK

Address for correspondence: Antje Frey Nascimento, M.Sc., Division of Clinical Psychology and Psychotherapy, Department of Psychology, University of Basel, Missionsstrasse 62a, 4055 Basel, Switzerland. Email: antje.freynascimento@unibas.ch; phone: +41 61 207 05 98.

For peer review only

ABSTRACT

Background: Recent evidence suggests that for certain clinical conditions, placebos can improve clinical outcomes even without deception. These so-called *open-label placebos* (OLPs) bear the advantage of a significant lower risk of adverse events and comply with ethical principles. Although premenstrual syndrome (PMS) seems to be considerably susceptible to placebo effects, no study has examined open-label placebo responses on PMS.

Methods and analysis: To test the efficacy of OLPs in women suffering from PMS, a clinical randomised controlled trial was designed to investigate on the effect on PMS. PMS symptoms are monitored on a daily basis via a symptom diary, adverse events are monitored intermittently. The study started in spring 2018 and patients will be included until a maximum of 150 participants are randomized. Besides PMS symptom intensity and interference, an array of further variables are assessed.

Ethics and dissemination: Ethics approval was obtained by the local ethical committee. Results of the main analysis and of secondary analyses will be submitted for publication in peer-reviewed journals.

Trials registration number:

- ClinicalTrials.gov Identifier: NCT03547661; "Open-Label Placebo Treatment of Women with Premenstrual Syndrome (OLPPMS_1)", was registered on 6th of June, 2018
- Swiss national registration: SNCTP000002809; "Open-label Placebo Behandlung bei Frauen mit prämenstruellem Syndrom – eine randomisiert-kontrollierte Studie", was registered on 18th May, 2018

For peer review only

KEYWORDS

open-label placebos; premenstrual syndrome; randomized controlled trial; treatment rationale; PMS symptom diary

For peer review only

ARTICLE SUMMARY

Strengths and limitations of this study

- This is the first clinical randomized controlled trial to test an open-label placebo (OLP) intervention in PMS.
- Moreover, this trial focuses on OLP responses on a somatic and a psychological outcome.
- Enrolled women are comprehensively and prospectively screened for PMS and the primary outcomes also are prospectively assessed.
- We test the effect of a plausible, comprehensive OLP treatment rationale, which may be relevant for a better understanding of effects of treatment rationales in general.
- Since different definitions of PMS exist, we based our definition on the most highly recommended diagnosis assessment criteria and assessment tools.

For peer review only

INTRODUCTION

Recent evidence suggests that in certain clinical conditions - such as chronic low-back pain [1], irritable bowel syndrome [2], rhinitis [3], and cancer-related fatigue [4] - placebos improve clinical outcomes even without deception. These so-called *open-label placebos* (OLP) are prescribed with a plausible scientific treatment rationale, encompassing that placebos are powerful; the body automatically responds to the intake of placebos; a positive attitude towards the intervention can be helpful; and taking the placebo pills faithfully is critical [1 2 5]. We have shown in a standardized heat pain experiment that the provided treatment rationale is crucial for the elicitation of an OLP response [6]. To date, no study has examined OLP responses on premenstrual syndrome (PMS), although PMS appears to be susceptible to placebo effects [7-9]. Moreover, a further analysis of the effect of the OLP treatment rationale can inform about intervention-independent effects of treatment rationales. Hence, we set out to examine OLP responses in PMS and the relevance of a plausible treatment rationale.

RATIONALE

PMS entails clinically significant somatic and psychological symptoms in the premenstrual phase of the menstrual cycle, causing substantial distress and functional impairment [10]. Worldwide, a considerable amount of women of reproductive age are affected with a pooled prevalence of 47.8%[11]. Subsequently, a myriad of distinctive therapies are prescribed for PMS - including pharmacological and phytopharmaceutic drugs as well as complementary interventions - [12] yet there is limited evidence for some interventions (e.g., Chaste tree; anxiolytics) while other empirically validated interventions (e.g., SSRIs, hormonal therapy) show considerable side-effects[13].

For peer review only - http://bmjopen.bmj.com/site/about/guidelines.xhtml

Notably, PMS seems to be highly susceptible to placebo effects: The Royal College of Obstetricians and Gynaecologists (RCOG) alerts to substantial placebo responses in randomized-controlled PMS intervention trials of 36-43%[7] and different studies reported considerable placebo effects on PMS [8 9 14]. Furthermore, PMS offers the opportunity to investigate OLP effects on *both* somatic and psychological symptoms. In addition, we are the first to assess the effect of a comprehensive OLP treatment rationale in a clinical population. We hypothesize that first, women obtaining an OLP treatment with a treatment rationale will report less PMS symptom intensity and interference during the course of the intervention and at follow-up, compared to women obtaining an OLP treatment without a treatment rationale. Second, we assume that women receiving an OLP treatment without a treatment rationale will also show a higher decrease in PMS symptom intensity and interference in comparison to the treatment as usual group (TAU).

For peer review only

OBJECTIVES

Primary objectives

The primary objective of this study is to test the effectiveness of an OLP treatment in women suffering from moderate to severe PMS in regard to PMS symptom intensity and interference. Also, we examine whether there is a group difference across time. Moreover, we test whether a comprehensive OLP treatment rationale has an effect on the OLP response compared to omitting the treatment rationale.

Secondary objectives

1. To test whether an OLP response is observed for somatic or on psychological symptoms
2. To assess the effect of the OLP treatment on PMS impact
3. To evaluate the effect of the OLP intervention on quality of life
4. To assess whether there is any impact of elevated baseline anxiety or depression levels on the OLP response
5. To investigate whether there is any effect of the OLP intervention on relationship satisfaction
6. To assess whether positive attitudes towards complementary and alternative medicine are associated with OLP responses
7. To assess the impact of attitudes towards placebos on the OLP response
8. To evaluate whether the appraisal of the treatment provider has any effect in regard of the OLP effect
9. To evaluate the intervention credibility in the two intervention groups
10. To investigate whether expectancy of relief and desire for relief have an effect on the OLP response
11. To assess treatment adherence of placebo pill intake

For peer review only - http://bmjopen.bmj.com/site/about/guidelines.xhtml

Table 1

Overview about research questions and study design

Research questions:

- Does an open-label placebo (OLP) intervention alleviate premenstrual syndrome (PMS) symptoms over time?
- Do participants who receive a comprehensive OLP treatment rationale show a different OLP response in comparison to participants who do not receive any treatment rationale over time?

Population:

Women suffering from a moderate to severe PMS, aged between 18 and 45 with a regular menstrual cycle and no essential comorbidities.

Intervention groups:

There are two intervention groups: one OLP with a plausible treatment rationale (OLP+) and an OLP without a plausible treatment rationale (OLP-) group. Both groups obtain openly administered placebo pills, hence two pills per day for six weeks.

Comparison:

A treatment as usual (TAU) group without any intervention but the same number of study contacts as the intervention groups and the same study procedures serves as control group.

Outcome:

Primary endpoint are PMS symptom intensity and interference measured by means of a PMS symptom diary.

Time of study duration:

Approximately 2 years.

For peer review only - http://bmjopen.bmj.com/site/about/guidelines.xhtml

METHODS AND ANALYSIS

Study design

A single-centre, randomized controlled clinical trial of an OLP intervention on women with PMS using a parallel group between-subject design with three study groups: a TAU group; an OLP with a plausible treatment rationale (OLP+) and an OLP without a treatment rationale (OLP-) group. The first participant was enrolled and randomized in August 2018 and the study is expected to be concluded by spring 2020. The study is being conducted at the Division of Clinical Psychology and Psychotherapy at the Faculty for Psychology of the University of Basel and was approved by the local ethical committee. For an overview, please see Table 1. The design of the trial is summarized in Figure 1 and all steps and aspects are delineated in the following.

Subjects

In total, 150 women suffering from moderate to severe PMS will be randomly allocated to one of the three study groups.

Patient and public involvement statement

Previous studies about patient experiences in OLP and placebo trials of IBS were consulted for this study to include patient experiences [15].

Patient eligibility

Women aged between 18 and 45 years with a regular menstrual cycle, suffering from moderate to severe PMS reported by prospective self-report are eligible for study participation [16]. A moderate to severe PMS diagnosis and the primary outcomes are identically assessed and calculated (for more details, see *Primary outcome measures*). Moreover, women have to be capable of consent, fluent in German and

For peer review only - http://bmjopen.bmj.com/site/about/guidelines.xhtml

have a menstrual cycle duration ranging from 24 to 31 days. The severity of PMS must warrant the treatment of at least one functionally impairing physical or psychological symptom based on the women's self-report [10]. Also, participants have to know a GP or gynaecologist who they would consult if necessary. The recent initiation of a new medication (i.e., within the last 30 days) leads to study exclusion or delayed enrolment, whereas the intake of any psychopharmacological or psychotropic substance or alcohol abuse lead to exclusion. Further exclusion criteria are pregnancy; breast-feeding; indications of a psychiatric or gynaecological disorder; suicidality; further drug intake affecting PMS symptoms; a BMI above 30; hypersensitivity or allergy to the placebo pills; participation in another study with drugs or in another PMS study within the last three months; and participation in psychotherapy due to PMS.

Recruitment

Participants are recruited via advertisement for "a novel efficacy study of an integrative and side-effect free intervention against premenstrual complaints" with flyers and posters at ambulant gynaecologists in the Region of Basel and in the gynaecological clinic of the University Hospital of Basel. Moreover, different internet platforms are used for advertisement and different public facilities and stores around Basel, including the German border region.

Potential participants are informed that participation in the study is voluntary, withdrawal of consent is possible at any time without mentioning reasons, and refusal of study participation will not lead to any negative consequences. Interested women obtain a study information form and all relevant information are also provided during the informed consent process with the opportunity to clarify questions. Women are only enrolled after written informed consent obtainment.

For peer review only - http://bmjopen.bmj.com/site/about/guidelines.xhtml

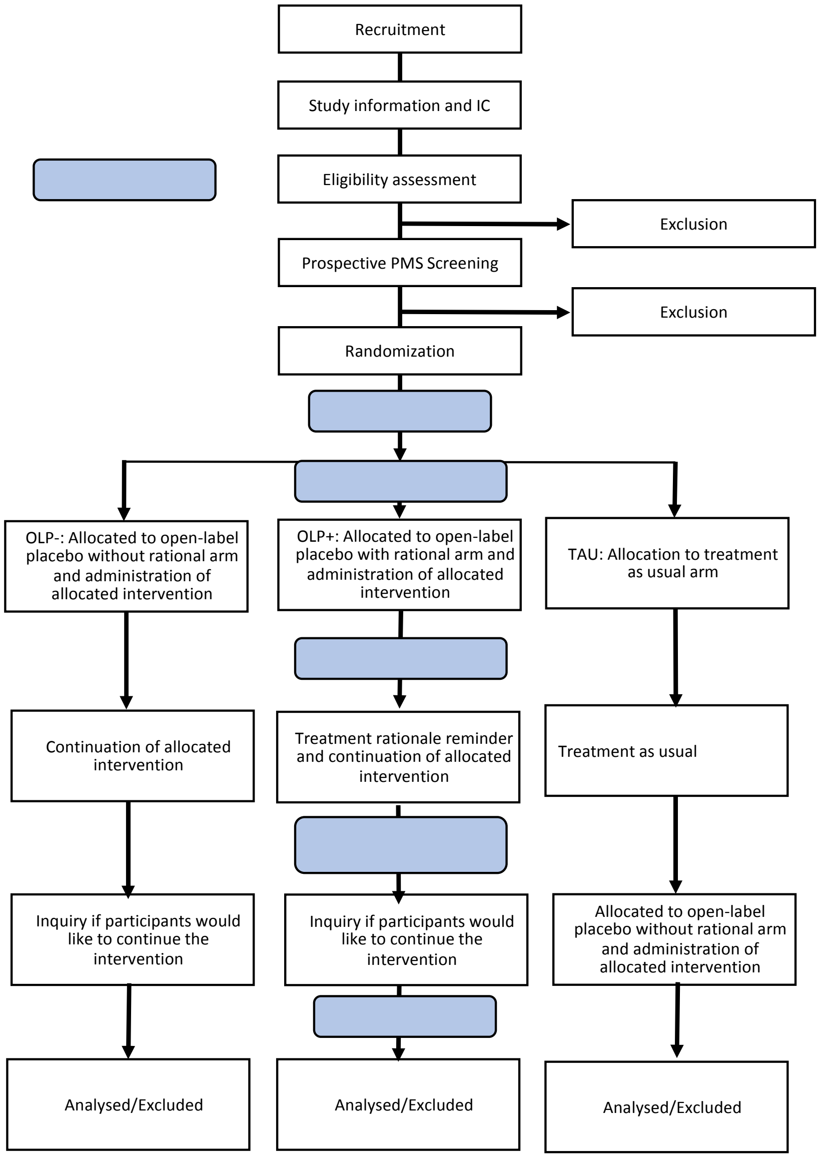

Figure 1. *Study Design and Flow of Participants*

Trial treatment and study arms

Following recruitment and verification of study eligibility - including a prospective PMS screening for one menstrual cycle - qualified women are allocated randomly by means of a random allocation sequence using the built-in random number generator in Microsoft Excel® to one of the three study groups: 1.) a TAU group; 2.) an OLP+ group, and 3.) an OLP- group (see also Figure 2).

1.) The TAU group serves as a control group and has the same amount of contacts with the study team. However, participants of this group do not receive any intervention. They are allowed to continue any medication intake, if the substance does not lead to study exclusion and PMS complaints are still prevalent. The same is warrant for both intervention groups.

2.) The OLP+ group obtains the intervention with a plausible treatment rationale. The intervention is provided at the personal meeting at the study site (t0), followed by a second study contact after three weeks by phone or if desired by participants again at the study site (t1) and a last posttreatment phone contact and assessment after the second treatment cycle (t2). The intervention consists of an administration of open-label placebo pills including the instruction to take two pills a day for six weeks. After the explicit mentioning of the pharmacological inertness of the pills in both groups, the OLP+ group receives - in contrast to the OLP- group - additionally a comprehensive OLP treatment rationale at t0 as well as at t1. The discussion points encompass: a) that placebos can be powerful, also for PMS; b) an explanation on why placebos work; c) that faithful pill intake is crucial and that doubts do not have a negative effect on the treatment; d) that placebos have also been shown to work without deception. Moreover, a video sequence of about 2 minutes is shown of an American NBC news report with German subtitle about an OLP inetrvention on irritable bowel syndrome including a patient report (see: https://www.youtube.com/watch?v=uv0SuWKZjsI&t=1s). Neither participants nor study team members are blinded. Yet besides the treatment provider, study team members are not informed about group allocation to minimise investigator bias. As investigational product, pink oral placebo pills are administered from the brand *Winthrop*, which are certified placebo pills with

For peer review only - http://bmjopen.bmj.com/site/about/guidelines.xhtml

validated quality. Also, participants of both intervention groups receive a certificate, confirming that the study medication only contains placebo pills.

3.) The OLP- group obtains the same intervention and intake instruction like the OLP+ group and the proceeding is identically. However, no treatment rationale or further treatment explanation is provided.

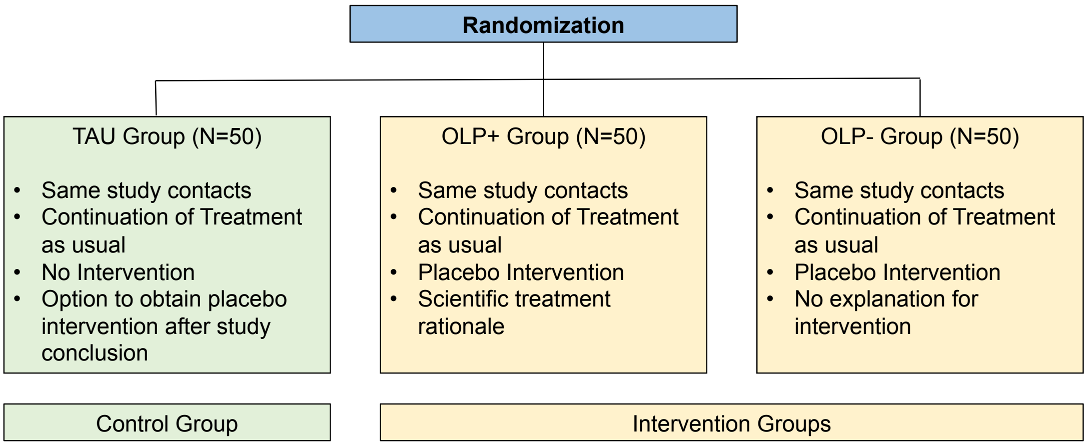

Figure 2. *Overview of Study Groups*

ew only

Detailed screening and PMS assessment

After informed consent obtainment, participants are comprehensively screened for study eligibility. Therefore, the following information is collected online:

- Verification of eligibility according to eligibility criteria
- Medical and gynaecological history
- Assessment of sociodemographic data
- Assessment of PMS retrospectively

In a second step, women fulfilling eligibility criteria and PMS criteria retrospectively, start a prospective PMS screening with daily online surveys as part of a PMS

For peer review only - http://bmjopen.bmj.com/site/about/guidelines.xhtml

symptom diary assessment for one menstrual cycle for the screening and in total for three menstrual cycle for primary outcome assessment (see below).

For peer review only

OUTCOME MEASURES: PRIMARY, SECONDARY, AND TERTIARY

Primary outcome measures

The German PMS symptom diary consists of 27 symptoms and of three further items assessing interference in different domains (i.e., work or school; social activities; relations with others). Usually, a four point Likert scale is employed, however, in line with Endicott et al. [17] we apply a six point Likert scale to be able to detect subtle changes. We compare changes over time of PMS symptom intensity and interference across three menstrual cycles. The *PMS symptom intensity score* is derived from the first 27 items of the German PMS symptom diary, and the *symptom interference score* is derived from the last three items. With regard to symptom intensity, two additional subscales are calculated by dividing the 27 symptom items into a psychological symptom intensity subscale and a physical symptom intensity subscale score (see secondary outcomes) [17].

Following Kues et al. [18] and Janda et al. [16], we calculate separate scores for the luteal and follicular phase and consider the difference for our analysis. In more detail, we conservatively consider 7 days prior to menstruation onset as a timeslot for the luteal phase and 7 days of the follicular phase, whereas the start of the follicular phase for each participant is calculated using the following formula: (individual cycle duration in days-14)/2 = first day of the follicular phase. Subsequently, we calculate the difference score of the means for each menstrual cycle for the respective primary outcome of interest (i.e., for PMS symptom intensity or interference). Intensity and interference are considered as marked if rated by participants with at least a 4 (very strong) on a scale from 0 (not at all) to 5 (extreme). Moreover, the item has to be rated with at least a four for at least two consecutive days to be considered as marked. Further, symptoms only are considered as marked symptoms if the respective symptom is not marked in the follicular phase and if it is associated with

For peer review only - http://bmjopen.bmj.com/site/about/guidelines.xhtml

marked interference in the luteal phase. If a participant has four or more symptoms marked in the luteal phase, also interference of only one day is accepted. We are also interested in the trajectory of the symptoms in the intervention groups across the two luteal phases after t0. Hence, we will compare if there are differences of group effects at cycle 2 (C2) and cycle 3 (C3) in contrast to cycle 1 (C1).

Moreover, we evaluate the effect of a comprehensive OLP treatment rationale, by providing one intervention group with a comprehensive treatment rationale in addition to intake instructions whereas the other intervention group only obtains intake instructions (see also *Trial treatment and study arms*).

Secondary outcome measures

Secondary outcomes comprise the following:

- Physical and psychological symptom subscale of the Ger*man PMS Symptom Diary* [16]*:*

  To test whether an OLP response occurs primarily on physical or on psychological symptoms, the above-mentioned German PMS Symptom Diary is used including the aforementioned calculation methods. The following scores are derived from the diary data of three menstrual cycles:

  - Physical symptom subscore: 14 items of the German PMS symptom diary are considered: depressed mood; hopelessness; feeling of worthlessness; feeling anxious; feeling tensed or being stressed out; irritability; sudden attacks of sadness; sudden crying; sensitivity to rejections; feeling angry; controversies with others; loss of interest in common activities; impaired concentration; feeling out of control or overwhelmed.

For peer review only

- Psychological symptom subscore: The following seven items are considered: Breast tenderness; breast sensitivity; breast swelling; headache; joint pains; muscle pain; feeling of a general body swelling; weight gain.

- *PMS-Impact Questionnaire* (PMS-I) [19]:

  The PMS-I measures the impact of premenstrual syndromes with 18 items and provides a total score and two subscales - a psychological impact and a functional impact scale. It was found to be a valid, reliable (Cronbach's alpha = .90) and economic assessment tool of PMS impact [16]. The questionnaire differentiates between functional and psychological impact [19]. In comparison to the German PMS symptom diary, it covers more domains of daily life (e.g., sexual experiences, physical exertion, stress coping) and is more specific for the detection of small changes of PMS impact. The PMS-I is assessed three times during the luteal phase, once during the prospective screening (L1) and twice during the intervention phase (L2 & L3).

- *Short Form-12 Health Survey for health-related quality of life* (SF-12) [20]:

  The SF-12 measures health-related quality of life independent of disorders, comprising 12 items, providing a physical and mental component summary scale. Health-related quality of life is assessed since PMS symptoms have a high impact on women's quality of life [21]. Physical health is comprised of the dimensions of physical functioning, physical role-functioning, bodily pain, general health; mental health is comprised of the dimensions of vitality, social functioning, emotional role-functioning, and mental health. The SF-12 is a validated and economic version of the SF36. For our purposes, we ask for ratings of the last week including today (premenstrual phase). The SF-12 is

For peer review only - http://bmjopen.bmj.com/site/about/guidelines.xhtml

completed three times by participants: during the luteal phase of the prospective screening (L1) and twice during the intervention phase (L2 & L3).

- *Hospital Anxiety and Depression Scale – German Version* (HADS-D) [22]:
  The HADS-D aims to assess anxiety and depression in individuals with somatic conditions and can be used for self-reported severity evaluation. It comprises two subscales (anxiety and depression) with seven items each and with a four-level answer format. The sum score indicates general psychological interference. We decided to use the HADS-D since it is a very sensitive tool for less severe cases of psychological disorders, which is usually the case for patients in a primarily somatic setting. We assess the HADS-D only at L1 during the first luteal phase before the start of the intervention.
- *A scale for assessment of satisfaction in close relationships* (ZIP) [23]:
  The ZIP is the German version of the relationship assessment scale by Hendrick et al. [24] and measures satisfaction in romantic relationships. The questionnaire entails seven items and had been shown to have high reliability and a clear unifactorial structure [24]. The ZIP is rated three times by participants during the luteal phases of the prospective screening (L1) and twice during the intervention phase (L2 & L3).
- Treatment adherence is measured by counting the remaining pills:
  Participants are asked to return the blister and pill boxes after the intervention.
- Expectancy of relief and desire for relief of premenstrual symptom severity are assessed at t0 by means of a Likert scale ranging from 1-6, like the primary outcome measure (German PMS Symptom Diary) and three weeks after intervention start.
- *Complementary and Alternative Medicine Beliefs Inventory* (CAMBI) [25]:

For peer review only

The CAMBI serves to assess three different aspects of complementary alternative medicine intervention beliefs with a 17-item questionnaire with satisfactory validity and reliability measures. Three dimensions comprise beliefs in natural treatments, participation in treatment, and holistic health. Participants fill out the inventory posttreatment at t2.

- Item about placebo appraisal of the *Questionnaire on Responders' Attitude Regarding Non-Specific Therapies* [26]:

  At t2, participants rate the item about placebo appraisal online which encompasses the following three questions:

  1) How would you define the term placebo?

  2) A rating of the positivity or negativity of the term.

  3) Do you believe that physical complaints can get better if you merely belief in the efficiency of a therapy?

- Patient-provider connection items of the *Healing Encounters and Attitudes Lists – German Short Version* (HEAL-D-SF) [27]: The HEAL-S provides an item bank to measure nonspecific factors in treatment. We collect the patient-provider connection item at t2 to control for the influence of provider perception.

- Placebo intervention credibility is assessed online at t2 with the question:

  Did you believe that you received placebo pills during the study, i.e. pills without any pharmacological content? The three answer options are:

  1) Yes, I was sure that I received placebo pills.

  2) I had doubts that I received placebo pills

  3) No, I did not believe that I received placebo pills.

  Moreover, the OLP- group is asked whether they felt something was missing, whereas the OLP+ group is asked whether they found the provided rationale convincing and how it may have helped or confused them personally.

For peer review only - http://bmjopen.bmj.com/site/about/guidelines.xhtml

Tertiary outcomes measures

Other, tertiary outcomes of interest are:

- Sociodemographic variables: age, family and relationship status, sexual orientation, citizenship, country of birth, mother tongue, educational degree, employment status, height, weight, and number of births and aborts, if participant has a GP/gynaecologist.
- Retrospective PMS Questionnaire sum score
- Mini-International Neuropsychiatric Interview (MINI)
- Pregnancy test
- Medical and gynaecological history
- Concomitant pharmacological or non-pharmacological interventions and changes in all groups
- Qualitative answers regarding treatment and study experience
- Occurrence of side-effects/adverse events
- Number of participants who withdraw from study participation
- Number of participants from the TAU group who attend an open-label placebo intervention after study conclusion
- Number of participants from the intervention groups who show interest to continue the open-label placebo intervention after study conclusion
- Suicidality

Adverse events and safety monitoring

Study participants will be asked about any side-effects, including also treatment-emergent adverse-events (TEAEs) or serious adverse events (SAEs) at t1 and t2. Suicidality, which is specifically associated with premenstrual dysphoric disorders [28], and generally found to be higher in the luteal (=premenstrual) phase [29], will also be assessed at each study visit and if necessary, safety actions will be provided.

For peer review only - http://bmjopen.bmj.com/site/about/guidelines.xhtml

SAMPLE SIZE AND STATISTICAL ANALYSIS OF DATA

Data management

For data management, Microsoft Excel® and LabKey®, which is hosted by the University of Basel, is used. Study data is collected at the web server-based software LimeSurvey®. Only authorized study team members are able to view and export data. Entries and actions are marked with the initials of the respective study member.

Sample size

A calculation with a power analysis on the basis of an F-test and an ANCOVA for three groups, using the statistical software G*Power, indicated a total sample size of 206 participants needed for a power of 0.9 and a total sample size of 158 participants for a power of 0.8 to detect a medium effect size of f=0.25 (equals d=0.5) with an alpha-level of 0.05.

Since we will adopt a multilevel approach which enables more precise estimations, we also conducted a power analysis on a repeated-measures ANOVA, using G*Power, whereby we are interested in the interaction between time (menstrual cycles, 3 levels) and study groups (3 levels). If the correlation for the outcome among the different time points (1-3 cycles) is assumed to be 0.5, then based on an alpha-level of 0.05, a power of 0.8, and a medium effect size of f=0.25, the required samples size is 36 (12 per group). Accordingly, for the main effect of menstrual cycle, we would need 30 participants (10 per group), and for the main effect of study group we would need 108 participants (36 per group). On this basis, we chose a conservative total sample size of 150 participants (50 per group). It is worth noting that most open-label placebo studies reported effect sizes that are generally higher than d=.5 (e.g., chronic low back pain: d=.77 [1]; irritable bowel syndrome: d=.79 [2]). However, in another open-label placebo study for patients suffering from major

For peer review only

depressive disorder [5], the authors reported an effect size of d=.54 whilst stating that the study was limited by low statistical power (i.e., the findings did not support the hypothesis that open-label placebos are effective in depression). Since PMS is not only characterised by physical complaints, yet also by psychological symptoms such as depressive mood, and since there is no specific research on open-label placebo effects and effect sizes in PMS, we considered a conservative medium effect size as more appropriate. This resulted in a more conservative sample size.

Statistical analysis

For the testing of the primary hypotheses, the daily data from three menstrual cycles of a symptom diary is examined. Because of the hierarchical structure of the data (menstrual cycles as level-1 are nested within participants as level-2) and given our aim to predict post-interventional changes between menstrual cycles as the primary outcome, a multilevel modelling approach will be adopted. Multilevel models take care of the interdependence of the hierarchical data that arises due to the fact that observations within the same individual are typically more similar to each other compared to observations between individuals [30 31]. Here, we will analyse temporal changes across menstrual cycles of symptom intensity and interference among the different study groups. To compare a TAU group with two intervention groups and to evaluate the effect of the applied treatment rationale, the following linear *a priori* contrast will be tested for the factor study group: TAU < OLP- < OLP+. Thus, based on published findings [6], we expect that the effect of OLP- lies between that of TAU and OLP+. Out statistical model contains menstrual cycle as within-subject factor with three levels (C1, C2, and C3), study group as between-subject factor with three levels (TAU, OLP+, and OLP–), and the interaction between the two factors. In line with recommendations by Fitzmaurice et al.[32], we will treat baseline values (i.e.,

For peer review only - http://bmjopen.bmj.com/site/about/guidelines.xhtml

values at C1) as a part of the outcome vector and assume that the means among study groups are equal at baseline since participants are randomized. The model contains the two main effects study group contrast (defined above) and menstrual cycle (temporal effect) plus the interaction between the two. We will be primarily interested in the interaction term as it tests for the differential temporal change between study groups in order as defined in the linear contrast.

Analyses of secondary outcomes are based on the same multilevel model as used for primary outcomes, but will be considered exploratively, thereby not correcting for multiple tests [33].

Monitoring

The study is monitored for quality and regulatory adherence. The monitor, who is not involved in our study and approved by the local ethical committee, verifies the qualification of the investigators and study team members, and monitors sound and appropriate documentation.

Ethics and dissemination

The present protocol and applied informed consent forms were approved with regard to their content and compliance with ethical regulations by the *Ethics Committee for Northwest/Central Switzerland*. The study is carried out with principles enunciated in the current version of the Declaration of Helsinki [34] and the guidelines of Good Clinical Practice (GCP) issued by ICH [35].

Women interested in study participation receive a participant information sheet and consent form, describing the study and providing sufficient information for an informed decision about participation and data confidentiality and detailed oral information is provided. The results of the planned analyses will be published in a peer-reviewed journal.

DISCUSSION

For peer review only - http://bmjopen.bmj.com/site/about/guidelines.xhtml

Given the huge burden of women suffering from PMS [11 36], it is necessary to optimize treatment options and to enhance the understanding of intra-interventional processes in PMS treatment. Moreover, interventions - including medical or psychotherapeutic treatment - can generally benefit from an increased comprehension of treatment context-factors and related meaning responses [37 38] involved in ailment amelioration. We therefore address the investigation of PMS-interventional context-factors and OLP effects and mechanisms with our study and strive to contribute to further knowledge in both areas. Previous RCTs of PMS interventions indicate high placebo responses [7]. The presented trial is essential to test whether an OLP response can also be elicited in PMS and whether an amelioration of symptoms occurs primarily on a somatic or psychological level. Additionally, we assess the importance of a comprehensive treatment rationale in a clinical population independent of a specific intervention.

So far there have been different aspects of previous OLP studies which have been critically evaluated in a meta-analysis of clinical OLP studies [39]. For instance, a lack of an adequate control group in OLP trials has been pointed out. To address this issue, the TAU control group of our trial has the same amount of contact with the study staff as the two intervention groups. Furthermore, we added an OLP- group to test the effect of a comprehensive OLP treatment rationale in a clinical population. Hence, we aim to assess whether placebo pills alone are sufficient or whether a comprehensive treatment rationale is vital to elicit an OLP response and, thus, strive to distinguish OLP effects from positive framing [39].

Moreover, to tackle previous critique of potential researcher bias and lack of blinding [39], any interactions with participants regarding the study proceedings, including data collection, are conducted by study team members who do not provide the intervention and who are not informed about study group allocation at the study visit.

The intervention is provided by one person only (A.F.N.) who is not involved in any interactions related to data collection. Furthermore, we decided to not provide the treatment rationale to all participants, primarily to test the effect of the treatment rationale and to avoid any experiences of disappointment in the TAU control group. A further advantage of our study is the large planned sample of 150 participants, tackling a further previously mentioned critique point [39].

Because we are interested in understanding mechanisms of OLP responses, we assess an array of different questionnaires including attitudes and experiences of participation in the different study groups. In this sense, we aim to address different aspects of former criticism with our study design and to enhance knowledge about OLP effects.

To sum up, a positive OLP response in PMS would form the basis for future studies investing how to successfully harness placebo effects, in an ethical fashion, in clinical practice and to optimize PMS interventions that often entail side-effects.

For peer review only

For peer review only - http://bmjopen.bmj.com/site/about/guidelines.xhtml

AUTHOR CONTRIBUTIONS

All of the following authors contributed significantly to the conception of the presented study and to the manuscript: Antje Frey Nascimento (email: antje freynascimento@unibas.ch), Jens Gaab (email: jens.gaab@unibas.ch), Irving Kirsch (email: irvkirsch@gmx.de), Joe Kossowsky (email: joe.kossowsky@unibas.ch), Andrea Meyer (email: andrea.meyer@unibas.ch), Cosima Locher (email: cosima.locher@unibas.ch)

FUNDING

This study is supported by the Swiss National Science Foundation (Prof. Dr. Jens Gaab), grant number: 325130_170117, under the name “Taking the placebo further: open placebo”. Cosima Locher, PhD, received funding for this project from the Swiss National Science Foundation (SNSF): P400PS_180730 (Title: Overcoming Classificatory and Methodological Hurdles to Improve Treatment of Chronic Primary Pain: A Network Meta-Analytic Approach)

COMPETING INTERESTS

There are no known potential conflicts of interest.

ETHICAL APPROVAL

This study has been approved by the EKNZ Ethical Committee (ID 2017-02186; Open-label placebo treatment of women with premenstrual syndrome: A randomized controlled trial)

WORD COUNT

The manuscript entails 4572 words.

For peer review only

REFERENCES

1. Carvalho C, Caetano JM, Cunha L, et al. Open-label placebo treatment in chronic low back pain: a randomized controlled trial. *Pain* 2016;157(12):2766.
2. Kaptchuk TJ, Friedlander E, Kelley JM, et al. Placebos without deception: a randomized controlled trial in irritable bowel syndrome. *PLoS One* 2010;5(12):e15591. doi: 10.1371/journal.pone.0015591
3. Schaefer M, Harke R, Denke C. Open-Label Placebos Improve Symptoms in Allergic Rhinitis: A Randomized Controlled Trial. *Psychother Psychosom* 2016;85(6):373-74. doi: 10.1159/000447242
4. Hoenemeyer TW, Kaptchuk TJ, Mehta TS, et al. Open-label placebo treatment for cancer-related fatigue: a randomized-controlled clinical trial. *Scientific reports* 2018;8(1):2784.
5. Kelley JM, Kaptchuk TJ, Cusin C, et al. Open-label placebo for major depressive disorder: a pilot randomized controlled trial. *Psychother Psychosom* 2012;81(5):312-4. doi: 10.1159/000337053
6. Locher C, Nascimento AF, Kirsch I, et al. Is the rationale more important than deception? A randomized controlled trial of openlabel placebo analgesia. *Pain* 2017
7. Green L, O'Brien P, Panay N, et al. Management of Premenstrual Syndrome: Green-top Guideline No. 48. *BJOG* 2017;124(3):e73-e105. doi: 10.1111/1471-0528.14260
8. Van Ree JM, Schagen Van Leeuwen JH, Koppeschaar HP, et al. Unexpected placebo response in premenstrual dysphoric disorder: implication of endogenous opioids. *Psychopharmacology (Berl)* 2005;182(2):318-9. doi: 10.1007/s00213-005-0090-8
9. Sampson GA. Premenstrual syndrome: a double-blind controlled trial of progesterone and placebo. *The British Journal of Psychiatry* 1979;135(3):209-15.
10. Yonkers KA, O'Brien PMS, Eriksson E. Premenstrual syndrome. *The Lancet* 2008;371(9619):1200-10. doi: 10.1016/s0140-6736(08)60527-9
11. Direkvand-Moghadam A, Sayehmiri K, Delpisheh A, et al. Epidemiology of Premenstrual Syndrome (PMS)-A systematic review and meta-analysis study. *Journal of clinical and diagnostic research: JCDR* 2014;8(2):106.
12. Maharaj S, Trevino K. A Comprehensive Review of Treatment Options for Premenstrual Syndrome and Premenstrual Dysphoric Disorder. *J Psychiatr Pract* 2015;21(5):334-50. doi: 10.1097/PRA.0000000000000099
13. O'Brien PS, Rapkin A, Schmidt PJ. The premenstrual syndromes: PMS and PMDD: CRC Press 2007.
14. Freeman EW, Rickels K. Characteristics of placebo responses in medical treatment of premenstrual syndrome. *American Journal of Psychiatry* 1999;156(9):1403-08.
15. Kaptchuk TJ, Shaw J, Kerr CE, et al. "Maybe I made up the whole thing": placebos and patients' experiences in a randomized controlled trial. *Culture, Medicine, and Psychiatry* 2009;33(3):382-411.
16. Janda CP, Kues JNP, Andersson GP, et al. A symptom diary to assess severe premenstrual syndrome and premenstrual dysphoric disorder. *Women Health* 2016:1-18. doi: 10.1080/03630242.2016.1206055
17. Endicott J, Nee J, Harrison W. Daily Record of Severity of Problems (DRSP): reliability and validity. *Arch Womens Ment Health* 2006;9(1):41-9. doi: 10.1007/s00737-005-0103-y
18. Kues JN, Janda C, Kleinstäuber M, et al. Internet-based cognitive behavioural self-help for premenstrual syndrome: study protocol for a randomised controlled trial. *Trials* 2014;15(1):472.

For peer review only

19. Kues JN, Janda C, Kleinstauber M, et al. How to measure the impact of premenstrual symptoms? Development and validation of the German PMS-Impact Questionnaire. *Women Health* 2016;56(7):807-26. doi: 10.1080/03630242.2015.1118734
20. Ware Jr JE, Kosinski M, Keller SD. A 12-Item Short-Form Health Survey: construction of scales and preliminary tests of reliability and validity. *Medical care* 1996;34(3):220-33.
21. Nevatte T, O'Brien PM, Backstrom T, et al. ISPMD consensus on the management of premenstrual disorders. *Arch Womens Ment Health* 2013;16(4):279-91. doi: 10.1007/s00737-013-0346-y
22. Herrmann C, Buss U, Snaith R. HADS-D: hospital anxiety and depression scale-German version. *Bern: Hans Huber* 1995;1:995.
23. Hassebrauck M. ZIP–Ein Instrumentarium zur Erfassung der Zufriedenheit in Paarbeziehungen. *Zeitschrift für Sozialpsychologie* 1991;22(4):256-59.
24. Hendrick SS, Dicke A, Hendrick C. The relationship assessment scale. *Journal of Social and Personal Relationships* 1998;15(1):137-42.
25. Bishop FL, Yardley L, Lewith G. Developing a measure of treatment beliefs: the complementary and alternative medicine beliefs inventory. *Complement Ther Med* 2005;13(2):144-9. doi: 10.1016/j.ctim.2005.01.005
26. Faessler M, Gnadinger M, Rosemann T, et al. Placebo interventions in practice: a questionnaire survey on the attitudes of patients and physicians. *Br J Gen Pract* 2011;61(583):101-7. doi: 10.3399/bjgp11X556209
27. Greco CM, Yu L, Johnston KL, et al. Measuring nonspecific factors in treatment: item banks that assess the healthcare experience and attitudes from the patient's perspective. *Quality of Life Research* 2016;25(7):1625-34.
28. Pilver CE, Libby DJ, Hoff RA. Premenstrual dysphoric disorder as a correlate of suicidal ideation, plans, and attempts among a nationally representative sample. *Soc Psychiatry Psychiatr Epidemiol* 2013;48(3):437-46. doi: 10.1007/s00127-012-0548-z
29. Saunders KE, Hawton K. Suicidal behaviour and the menstrual cycle. *Psychol Med* 2006;36(7):901-12. doi: 10.1017/S0033291706007392
30. Kalmbach DA, Kingsberg SA, Ciesla JA. How changes in depression and anxiety symptoms correspond to variations in female sexual response in a nonclinical sample of young women: a daily diary study. *The journal of sexual medicine* 2014;11(12):2915-27.
31. Field A, Miles J, Field Z. Discovering statistics using R: Sage publications 2012.
32. Fitzmaurice GM, Laird NM, Ware JH. Applied longitudinal analysis: John Wiley & Sons 2012.
33. Streiner DL, Norman GR. Correction for multiple testing: is there a resolution? *Chest* 2011;140(1):16-18.
34. WMA. World Medical Association Declaration of Helsinki: Ethical Principles for Medical Research Involving Human Subjects. *JAMA* 2013;310(20):2191-4. doi: doi: 10.1001/jama.2013.281053
35. ICH. International Conference on Harmonization-E6 Guideline for Good Clinical Practice 1996 [Available from: http://www.ich.org/fileadmin/Public_Web_Site/ICH_Products/Guidelines/Efficacy/E6/E6_R2__Step_4.pdf.
36. Halbreich U, Borenstein J, Pearlstein T, et al. The prevalence, impairment, impact, and burden of premenstrual dysphoric disorder (PMS/PMDD). *Psychoneuroendocrinology* 2003;28:1-23. doi: 10.1016/s0306-4530(03)00098-2

For peer review only - http://bmjopen.bmj.com/site/about/guidelines.xhtml

37. Moerman DE, Jonas WB. Deconstructing the placebo effect and finding the meaning response. *Annals of Internal medicine* 2002;136(6):471-76.
38. Annoni M, Blease C. A Critical (and Cautiously Optimistic) Appraisal of Moerman's "Meaning Response". *Perspect Biol Med* 2018;61(3):379-87. doi: 10.1353/pbm.2018.0050
39. Charlesworth JEG, Petkovic G, Kelley JM, et al. Effects of placebos without deception compared with no treatment: A systematic review and meta-analysis. *J Evid Based Med* 2017;10(2):97-107. doi: 10.1111/jebm.12251

For peer review only

STUDY PROTOCOL

OPEN-LABEL PLACEBO TREATMENT OF WOMEN WITH PREMENSTRUAL SYNDROME: STUDY PROTOCOL OF A RANDOMIZED CONTROLLED TRIAL

Figures

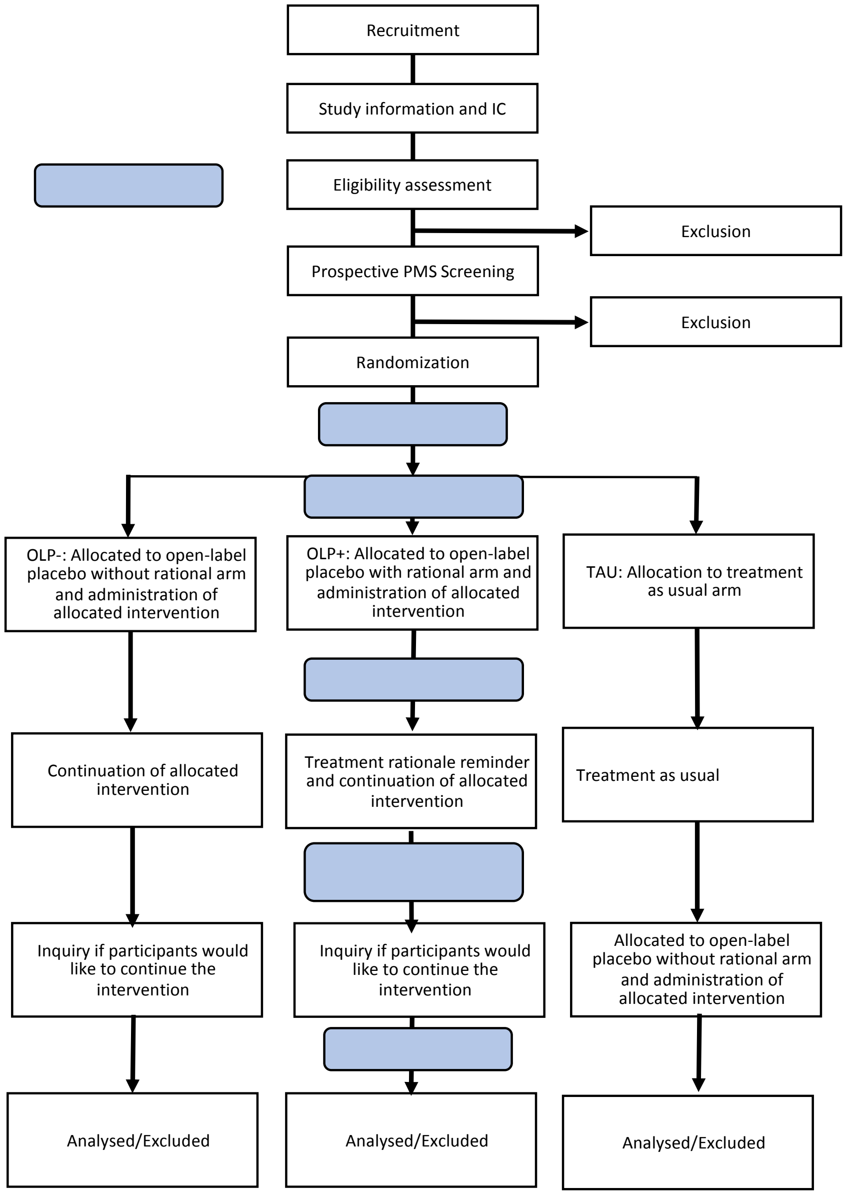

Figure 1. *Study Design and Flow of Participants*

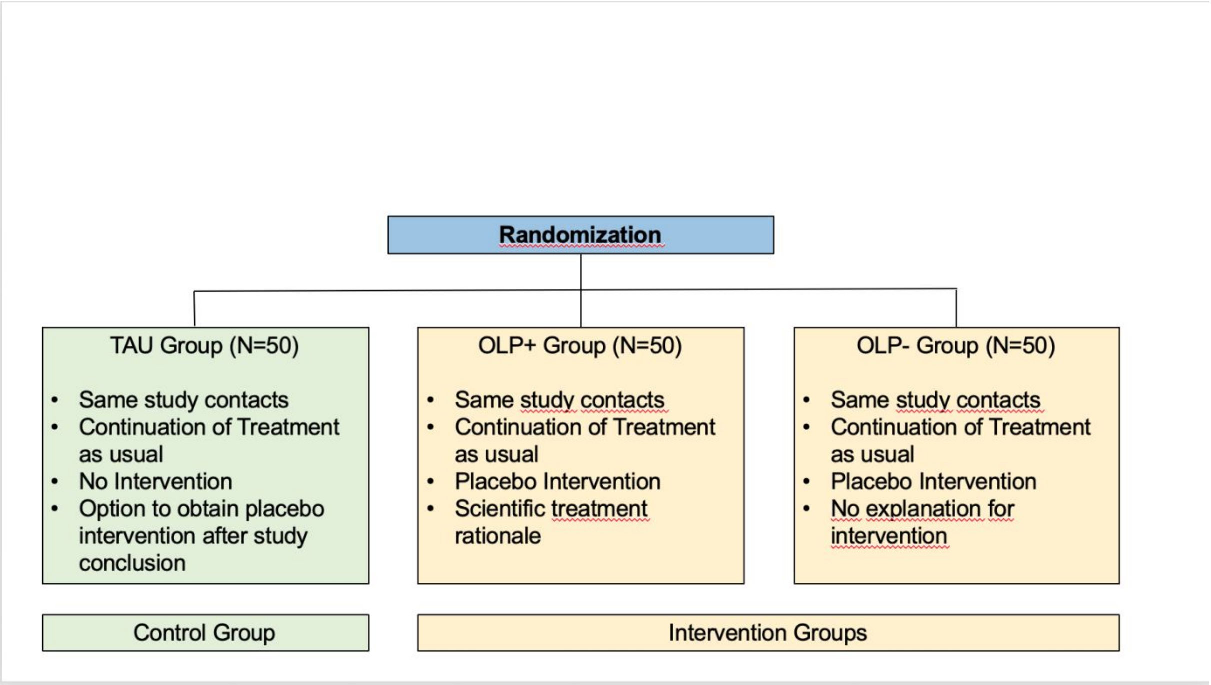

Figure 2. *Overview of Study Groups*

Reporting checklist for protocol of a clinical trial.

Based on the SPIRIT guidelines.

Instructions to authors

Complete this checklist by entering the page numbers from your manuscript where readers will find each of the items listed below.

Your article may not currently address all the items on the checklist. Please modify your text to include the missing information. If you are certain that an item does not apply, please write "n/a" and provide a short explanation.

Upload your completed checklist as an extra file when you submit to a journal.

In your methods section, say that you used the SPIRITreporting guidelines, and cite them as:

Chan A-W, Tetzlaff JM, Altman DG, Laupacis A, Gøtzsche PC, Krleža-Jerić K, Hróbjartsson A, Mann H, Dickersin K, Berlin J, Doré C, Parulekar W, Summerskill W, Groves T, Schulz K, Sox H, Rockhold FW, Rennie D, Moher D. SPIRIT 2013 Statement: Defining standard protocol items for clinical trials. Ann Intern Med. 2013;158(3):200-207

		Reporting Item	Page Number
**Administrative information**			
Title	#1	Descriptive title identifying the study design, population, interventions, and, if applicable, trial acronym	1
Trial registration	#2a	Trial identifier and registry name. If not yet registered, name of intended registry	2
Trial registration: data set	#2b	All items from the World Health Organization Trial Registration Data Set	2
Protocol version	#3	Date and version identifier	2
Funding	#4	Sources and types of financial, material, and other support	26
Roles and responsibilities: contributorship	#5a	Names, affiliations, and roles of protocol contributors	1; 26

For peer review only

Roles and responsibilities: sponsor contact information	#5b	Name and contact information for the trial sponsor	n/a
Roles and responsibilities: sponsor and funder	#5c	Role of study sponsor and funders, if any, in study design; collection, management, analysis, and interpretation of data; writing of the report; and the decision to submit the report for publication, including whether they will have ultimate authority over any of these activities	26
Roles and responsibilities: committees	#5d	Composition, roles, and responsibilities of the coordinating centre, steering committee, endpoint adjudication committee, data management team, and other individuals or groups overseeing the trial, if applicable (see Item 21a for data monitoring committee)	26
**Introduction**			
Background and rationale	#6a	Description of research question and justification for undertaking the trial, including summary of relevant studies (published and unpublished) examining benefits and harms for each intervention	5
Background and rationale: choice of comparators	#6b	Explanation for choice of comparators	8; 12
Objectives	#7	Specific objectives or hypotheses	7
Trial design	#8	Description of trial design including type of trial (eg, parallel group, crossover, factorial, single group), allocation ratio, and framework (eg, superiority, equivalence, non-inferiority, exploratory)	9
**Methods: Participants, interventions, and outcomes**			
Study setting	#9	Description of study settings (eg, community clinic, academic hospital) and list of countries where data will be collected. Reference to where list of study sites can be obtained	9
Eligibility criteria	#10	Inclusion and exclusion criteria for participants. If applicable, eligibility criteria for study centres and individuals who will	9

For peer review only

		perform the interventions (eg, surgeons, psychotherapists)	
Interventions: description	#11a	Interventions for each group with sufficient detail to allow replication, including how and when they will be administered	11
Interventions: modifications	#11b	Criteria for discontinuing or modifying allocated interventions for a given trial participant (eg, drug dose change in response to harms, participant request, or improving / worsening disease)	n/a
Interventions: adherance	#11c	Strategies to improve adherence to intervention protocols, and any procedures for monitoring adherence (eg, drug tablet return; laboratory tests)	18; 23
Interventions: concomitant care	#11d	Relevant concomitant care and interventions that are permitted or prohibited during the trial	12
Outcomes	#12	Primary, secondary, and other outcomes, including the specific measurement variable (eg, systolic blood pressure), analysis metric (eg, change from baseline, final value, time to event), method of aggregation (eg, median, proportion), and time point for each outcome. Explanation of the clinical relevance of chosen efficacy and harm outcomes is strongly recommended	15 ff.
Participant timeline	#13	Time schedule of enrolment, interventions (including any run-ins and washouts), assessments, and visits for participants. A schematic diagram is highly recommended (see Figure)	11
Sample size	#14	Estimated number of participants needed to achieve study objectives and how it was determined, including clinical and statistical assumptions supporting any sample size calculations	21
Recruitment	#15	Strategies for achieving adequate participant enrolment to reach target sample size	10
**Methods: Assignment of interventions (for controlled trials)**			
Allocation: sequence generation	#16a	Method of generating the allocation sequence (eg, computer-generated random numbers), and list of any factors for stratification. To reduce predictability of a random sequence, details of any planned restriction (eg, blocking) should be provided in a separate document that is unavailable to those who enrol participants or assign interventions	11

For peer review only

Allocation concealment mechanism	#16b	Mechanism of implementing the allocation sequence (eg, central telephone; sequentially numbered, opaque, sealed envelopes), describing any steps to conceal the sequence until interventions are assigned	12
Allocation: implementation	#16c	Who will generate the allocation sequence, who will enrol participants, and who will assign participants to interventions	12
Blinding (masking)	#17a	Who will be blinded after assignment to interventions (eg, trial participants, care providers, outcome assessors, data analysts), and how	n/a
Blinding (masking): emergency unblinding	#17b	If blinded, circumstances under which unblinding is permissible, and procedure for revealing a participant's allocated intervention during the trial	n/a
**Methods: Data collection, management, and analysis**			
Data collection plan	#18a	Plans for assessment and collection of outcome, baseline, and other trial data, including any related processes to promote data quality (eg, duplicate measurements, training of assessors) and a description of study instruments (eg, questionnaires, laboratory tests) along with their reliability and validity, if known. Reference to where data collection forms can be found, if not in the protocol	13 ff.
Data collection plan: retention	#18b	Plans to promote participant retention and complete follow-up, including list of any outcome data to be collected for participants who discontinue or deviate from intervention protocols	n/a
Data management	#19	Plans for data entry, coding, security, and storage, including any related processes to promote data quality (eg, double data entry; range checks for data values). Reference to where details of data management procedures can be found, if not in the protocol	21
Statistics: outcomes	#20a	Statistical methods for analysing primary and secondary outcomes. Reference to where other details of the statistical analysis plan can be found, if not in the protocol	22
Statistics: additional analyses	#20b	Methods for any additional analyses (eg, subgroup and adjusted analyses)	22

For peer review only

Statistics: analysis population and missing data	#20c	Definition of analysis population relating to protocol non-adherence (eg, as randomised analysis), and any statistical methods to handle missing data (eg, multiple imputation)	22
**Methods: Monitoring**			
Data monitoring: formal committee	#21a	Composition of data monitoring committee (DMC); summary of its role and reporting structure; statement of whether it is independent from the sponsor and competing interests; and reference to where further details about its charter can be found, if not in the protocol. Alternatively, an explanation of why a DMC is not needed	23
Data monitoring: interim analysis	#21b	Description of any interim analyses and stopping guidelines, including who will have access to these interim results and make the final decision to terminate the trial	n/a
Harms	#22	Plans for collecting, assessing, reporting, and managing solicited and spontaneously reported adverse events and other unintended effects of trial interventions or trial conduct	20
Auditing	#23	Frequency and procedures for auditing trial conduct, if any, and whether the process will be independent from investigators and the sponsor	23
**Ethics and dissemination**			
Research ethics approval	#24	Plans for seeking research ethics committee / institutional review board (REC / IRB) approval	26
Protocol amendments	#25	Plans for communicating important protocol modifications (eg, changes to eligibility criteria, outcomes, analyses) to relevant parties (eg, investigators, REC / IRBs, trial participants, trial registries, journals, regulators)	n/a
Consent or assent	#26a	Who will obtain informed consent or assent from potential trial participants or authorised surrogates, and how (see Item 32)	23
Consent or assent: ancillary studies	#26b	Additional consent provisions for collection and use of participant data and biological specimens in ancillary studies, if applicable	n/a
Confidentiality	#27	How personal information about potential and enrolled participants will be collected, shared, and maintained in order to protect confidentiality before, during, and after the trial	23; 21

For peer review only

Declaration of interests	#28	Financial and other competing interests for principal investigators for the overall trial and each study site	26
Data access	#29	Statement of who will have access to the final trial dataset, and disclosure of contractual agreements that limit such access for investigators	n/a
Ancillary and post trial care	#30	Provisions, if any, for ancillary and post-trial care, and for compensation to those who suffer harm from trial participation	n/a
Dissemination policy: trial results	#31a	Plans for investigators and sponsor to communicate trial results to participants, healthcare professionals, the public, and other relevant groups (eg, via publication, reporting in results databases, or other data sharing arrangements), including any publication restrictions	2
Dissemination policy: authorship	#31b	Authorship eligibility guidelines and any intended use of professional writers	n/a
Dissemination policy: reproducible research	#31c	Plans, if any, for granting public access to the full protocol, participant-level dataset, and statistical code	n/a
**Appendices**			
Informed consent materials	#32	Model consent form and other related documentation given to participants and authorised surrogates	n/a
Biological specimens	#33	Plans for collection, laboratory evaluation, and storage of biological specimens for genetic or molecular analysis in the current trial and for future use in ancillary studies, if applicable	n/a

The SPIRIT checklist is distributed under the terms of the Creative Commons Attribution License CC-BY-ND 3.0. This checklist was completed on 09. July 2019 using https://www.goodreports.org/, a tool made by the EQUATOR Network in collaboration with Penelope.ai

For peer review only

OPEN-LABEL PLACEBO TREATMENT OF WOMEN WITH PREMENSTRUAL SYNDROME: STUDY PROTOCOL OF A RANDOMIZED CONTROLLED TRIAL

Journal:	*BMJ Open*
Manuscript ID	bmjopen-2019-032868.R1
Article Type:	Protocol
Date Submitted by the Author:	07-Nov-2019
Complete List of Authors:	Frey Nascimento, Antje; University of Basel<br>Gaab, Jens; University of Basel<br>Kirsch, Irving; Harvard Medical School<br>Kossowsky, Joe; University of Basel; Harvard Medical School<br>Meyer, Andrea; University of Basel<br>Locher, Cosima; University of Basel; University of Plymouth
**Primary Subject Heading**:	Obstetrics and gynaecology
Secondary Subject Heading:	Ethics, Evidence based practice, Patient-centred medicine
Keywords:	open-label placebos, premenstrual syndrome, randomized controlled trial, treatment rationale, PMS symptom diary

SCHOLARONE™ Manuscripts

STUDY PROTOCOL

OPEN-LABEL PLACEBO TREATMENT OF WOMEN WITH PREMENSTRUAL SYNDROME: STUDY PROTOCOL OF A RANDOMIZED CONTROLLED TRIAL

Antje Frey Nascimento[1], Jens Gaab[1], Irving Kirsch[2], Joe Kossowsky[1, 2, 3], Andrea Meyer[4], Cosima Locher[1, 5]

[1] Division of Clinical Psychology and Psychotherapy, Department of Psychology, University of Basel, Switzerland

[2] Program in Placebo Studies, Beth Israel Deaconess Medical Center, Harvard Medical School, Boston, MA, USA

[3] Department of Anesthesiology, Critical Care & Pain Medicine, Boston Children's Hospital, Harvard Medical School, Boston, MA, USA

[4] Division of Clinical Psychology and Epidemiology, Department of Psychology, University of Basel, Switzerland

[5] University of Plymouth; School of Psychology, UK

Address for correspondence: Antje Frey Nascimento, M.Sc., Division of Clinical Psychology and Psychotherapy, Department of Psychology, University of Basel, Missionsstrasse 62a, 4055 Basel, Switzerland. Email: antje.freynascimento@unibas.ch; phone: +41 61 207 05 98.

For peer review only - http://bmjopen.bmj.com/site/about/guidelines.xhtml

ABSTRACT

Introduction: Recent evidence suggests that for certain clinical conditions, placebos can improve clinical outcomes even without deception. These so-called *open-label placebos* (OLPs) bear the advantage of a significant lower risk of adverse events and comply with ethical principles. Although premenstrual syndrome (PMS) seems to be considerably susceptible to placebo effects, no study has examined open-label placebo responses on PMS.

Methods and analysis: To test the efficacy of OLPs in women suffering from PMS, a clinical randomised controlled trial including two OLP study groups (with and without treatment rationale) was designed to investigate on the effect on PMS. PMS symptoms are monitored on a daily basis via a symptom diary, adverse events are monitored intermittently. The study started in spring 2018 and patients will be included until a maximum of 150 participants are randomized. Besides the primary outcome PMS symptom intensity and interference, an array of further variables is assessed. Multilevel modelling will be used for data analyses.

Ethics and dissemination: Ethics approval was obtained from the *Ethics Committee for Northwest/Central Switzerland*. Results of the main analysis and of secondary analyses will be submitted for publication in peer-reviewed journals.

Trials registration number:

- ClinicalTrials.gov Identifier: NCT03547661; “Open-Label Placebo Treatment of Women with Premenstrual Syndrome (OLPPMS_1)”, was registered on 6th of June, 2018
- Swiss national registration: SNCTP000002809; “Open-label Placebo Behandlung bei Frauen mit prämenstruellem Syndrom – eine randomisiert-kontrollierte Studie”, was registered on 18th May, 2018

For peer review only - http://bmjopen.bmj.com/site/about/guidelines.xhtml

KEYWORDS

open-label placebos; premenstrual syndrome; randomized controlled trial; treatment rationale; PMS symptom diary

For peer review only

ARTICLE SUMMARY

Strengths and limitations of this study

- This is the first clinical randomized controlled trial to test an open-label placebo (OLP) intervention in PMS.
- Moreover, this trial focuses on OLP responses on a somatic and a psychological outcome.
- To tackle the challenge of variations in PMS complaints and menstrual cycle lengths within subjects, enrolled women are comprehensively and prospectively screened for PMS and the primary outcomes are also prospectively assessed.
- We test the effect of a plausible, comprehensive OLP treatment rationale, which may be relevant for a better understanding of effects of treatment rationales in general.
- Since different definitions of PMS exist, we based our definition on the most highly recommended diagnosis assessment criteria and assessment tools.

For peer review only

INTRODUCTION

Recent evidence suggests that in certain clinical conditions - such as chronic low-back pain [1], irritable bowel syndrome [2], rhinitis [3], and cancer-related fatigue [4] - placebos improve clinical outcomes even without deception. These so-called *open-label placebos* (OLP) are prescribed with a plausible scientific treatment rationale, encompassing that placebos are powerful; the body automatically responds to the intake of placebos; a positive attitude towards the intervention can be helpful; and taking the placebo pills faithfully is critical [1 2 5]. We have shown in a standardized heat pain experiment that the provided treatment rationale is crucial for the elicitation of an OLP response [6]. To date, no study has examined OLP responses on premenstrual syndrome (PMS), although PMS appears to be susceptible to placebo effects [7-9]. Moreover, a further analysis of the effect of the OLP treatment rationale can inform about intervention-independent effects of treatment rationales. Hence, we set out to examine OLP responses in PMS and the relevance of a plausible treatment rationale.

RATIONALE

PMS entails clinically significant somatic and psychological symptoms in the premenstrual phase of the menstrual cycle, causing substantial distress and functional impairment [10]. Worldwide, a considerable amount of women of reproductive age are affected with a pooled prevalence of 47.8%[11]. Subsequently, a myriad of distinctive therapies are prescribed for PMS - including pharmacological and phytopharmaceutic drugs as well as complementary interventions - [12] yet there is limited evidence for some interventions (e.g., Chaste tree; anxiolytics) while other empirically validated interventions (e.g., SSRIs, hormonal therapy) show considerable side-effects[13].

For peer review only - http://bmjopen.bmj.com/site/about/guidelines.xhtml

Notably, PMS seems to be highly susceptible to placebo effects: The Royal College of Obstetricians and Gynaecologists (RCOG) alerts to substantial placebo responses in randomized-controlled PMS intervention trials of 36-43%[7] and different studies reported considerable placebo effects on PMS [8 9 14]. Furthermore, PMS offers the opportunity to investigate OLP effects on *both* somatic and psychological symptoms since symptom diaries like the German PMS symptom diary inquire about physical (e.g., breast tenderness, headaches, joint and muscle pain) as well as psychological symptoms (e.g., depressed mood, hopelessness, irritability). In addition, we are the first to assess the effect of a comprehensive OLP treatment rationale in a clinical population. We hypothesize that first, women obtaining an OLP treatment with a treatment rationale will report less PMS symptom intensity and interference during the course of the intervention and at follow-up, compared to women obtaining an OLP treatment without a treatment rationale. Second, we assume that women receiving an OLP treatment without a treatment rationale will also show a higher decrease in PMS symptom intensity and interference in comparison to the treatment as usual group (TAU), which is not given any of the treatment-specific information that the OLP groups receive nor the rationale for the OLP treatment.

For peer review only

OBJECTIVES

Primary objectives

The primary objective of this study is to test the effectiveness of an OLP treatment in women suffering from moderate to severe PMS in regard to PMS symptom intensity and interference. Also, we examine whether there is a group difference across time. Moreover, we test whether a comprehensive OLP treatment rationale has an effect on the OLP response compared to omitting the treatment rationale.

For peer review only

Secondary objectives

1. To test whether an OLP response is observed for somatic or on psychological symptoms
2. To assess the effect of the OLP treatment on PMS impact
3. To evaluate the effect of the OLP intervention on quality of life
4. To assess whether there is any impact of elevated baseline anxiety or depression levels on the OLP response
5. To investigate whether there is any effect of the OLP intervention on relationship satisfaction
6. To assess whether positive attitudes towards complementary and alternative medicine are associated with OLP responses
7. To assess the impact of attitudes towards placebos on the OLP response
8. To evaluate whether the appraisal of the treatment provider has any effect in regard of the OLP effect
9. To evaluate the intervention credibility in the two intervention groups
10. To investigate whether expectancy of relief and desire for relief have an effect on the OLP response
11. To assess treatment adherence of placebo pill intake

For peer review only - http://bmjopen.bmj.com/site/about/guidelines.xhtml

Table 1

Overview about research questions and study design

Research questions:

- Does an open-label placebo (OLP) intervention alleviate premenstrual syndrome (PMS) symptoms over time?
- Do participants who receive a comprehensive OLP treatment rationale show a greater OLP response in comparison to participants who do not receive any treatment rationale over time?

Population:

Women suffering from a moderate to severe PMS, aged between 18 and 45 with a regular menstrual cycle and no essential comorbidities.

Intervention groups:

There are two intervention groups: one OLP with a plausible treatment rationale (OLP+) and an OLP without a plausible treatment rationale (OLP-) group. Both groups obtain openly administered placebo pills, hence two pills per day for six weeks.

Comparison:

A treatment as usual (TAU) group without any intervention but the same number of study contacts as the intervention groups and the same study procedures serves as control group.

Outcome:

Primary endpoint are PMS symptom intensity and interference measured by means of a PMS symptom diary.

Time of study duration:

Approximately 2 years.

For peer review only

METHODS AND ANALYSIS

Study design

A single-centre, randomized controlled clinical trial of an OLP intervention on women with PMS using a parallel group between-subject design with three study groups: a TAU group; an OLP with a plausible treatment rationale (OLP+) and an OLP without a treatment rationale (OLP-) group. The first participant was enrolled and randomized in August 2018 and the study is expected to be concluded by spring 2020 with the planned inclusion of 150 study participants. The study is being conducted at the Division of Clinical Psychology and Psychotherapy at the Faculty for Psychology of the University of Basel and was approved by the local ethical committee *Ethics Committee for Northwest/Central Switzerland* EKNZ (ID 2017-02186). For an overview, please see Table 1. The design of the trial is summarized in Figure 1 and all steps and aspects are delineated in the following (see also Table 2 for an overview of the schedule).

Subjects

In total, 150 women suffering from moderate to severe PMS will be randomly allocated to one of the three study groups.

Patient and public involvement statement

Previous studies about patient experiences in OLP and placebo trials of IBS were consulted for this study to include patient experiences [15].

For peer review only - http://bmjopen.bmj.com/site/about/guidelines.xhtml

Table 2

Study Schedule

**Study Periods**	**Screening Phase**			**Intervention Phase**				
	**Preliminary Screening of Eligibility**	**Prospective PMS Screening**	**L1 OA**	**Personal Meeting (t0)**	**L2 OA**	**Midpoint Study Contact**	**L3 OA**	**Posttreatment Study Contact & OA (t2)**
Visit/contact	phone/post/online	at home (diary form)	online	at study site	online	at study site or phone/Skype	online	phone + posttreatment online
Day of menstrual cycle (MC)	-31 - -1 of 1. MC	1. day of 1. MC until end of 1. MC	-5 - -1 of 2. MC	1-14 day of 2. MC	-5 - -1 of 3. MC	1-14 day of 3. MC	-5 - -1 of 4. MC	1-14 day of 4. MC
Menstrual phase	at any point	one whole MC	luteal	follicular	luteal	follicular	luteal	follicular
Time (min.)	20	2 daily for 1 MC	10	60	10	15	10	30
Study information/ Informed Consent	x							
In-/Exclusion Criteria	x	x		x				
Randomisation				x				
Placebo dragée intake in intervention arms				x	x	x	x	
Narrative for OLP+ group				x		x		
**Primary Outcome:**								
PMS symptom diary		x	x	x	x	x	x	
**Safety Variables:**			x	x	x		x	x
Suicidality assessment	x			x		x		x
Side-Effects assessment						x		x

Note: L = luteal phase; OA = Online assessment; PMS = premenstrual syndrome.

For peer review only

Patient eligibility

Women aged between 18 and 45 years with a regular menstrual cycle, suffering from moderate to severe PMS reported by prospective self-report are eligible for study participation [16]. A moderate to severe PMS diagnosis and the primary outcomes are identically assessed and calculated (for more details, see *Primary outcome measures*). Moreover, women have to be capable of consent, fluent in German and have a menstrual cycle duration ranging from 24 to 31 days. The severity of PMS must warrant the treatment of at least one functionally impairing physical or psychological symptom based on the women's self-report [10]. Also, participants have to know a GP or gynaecologist who they would consult if necessary. The recent initiation of a new medication (i.e., within the last 30 days) leads to study exclusion or delayed enrolment, whereas the intake of any psychopharmacological or psychotropic substance or alcohol abuse lead to exclusion. Further exclusion criteria are pregnancy; breast-feeding; indications of a psychiatric or gynaecological disorder; suicidality; further drug intake affecting PMS symptoms; a BMI above 30; hypersensitivity or allergy to the placebo pills; participation in another study with drugs or in another PMS study within the last three months; and participation in psychotherapy due to PMS.

Recruitment

Participants are recruited via advertisement for "a novel efficacy study of an integrative and side-effect free intervention against premenstrual complaints" with flyers and posters at ambulant gynaecologists in the Region of Basel and in the gynaecological clinic of the University Hospital of Basel. Moreover, different internet platforms are used for advertisement and different public facilities and stores around Basel, including the German border region.

For peer review only - http://bmjopen.bmj.com/site/about/guidelines.xhtml

Potential participants are informed that participation in the study is voluntary, withdrawal of consent is possible at any time without mentioning reasons, and refusal of study participation will not lead to any negative consequences. Interested women obtain a study information form (supplementary material 1-2) and all relevant information are also provided during the informed consent process with the opportunity to clarify questions. Women are only enrolled after written informed consent obtainment.

Trial treatment and study arms

Following recruitment and verification of study eligibility - including a prospective PMS screening for one menstrual cycle - qualified women are allocated randomly by means of a random allocation sequence using the built-in random number generator in Microsoft Excel® to one of the three study groups at the study visit (t0) and also are informed before any intervention, respectively the control contact starts: 1.) a TAU group; 2.) an OLP+ group, and 3.) an OLP- group (see also Figure 2).

1.) The TAU group serves as a control group and has the same amount of contacts with the study team. However, participants of this group do not receive any intervention nor any information concerning the intervention. However, they are told why the control group is valuable and essential for the trial and that they will receive the intervention after study conclusion if desired. They are allowed to continue any medication intake, if the substance does not lead to study exclusion and PMS complaints are still prevalent. The same is warrant for both intervention groups.

2.) The OLP+ group obtains the intervention with a plausible treatment rationale. The intervention is provided at the personal meeting at the study site (t0),

For peer review only - http://bmjopen.bmj.com/site/about/guidelines.xhtml

followed by a second study contact after three weeks by phone or if desired by participants again at the study site (t1) and a last posttreatment phone contact and assessment after the second treatment cycle (t2). The intervention consists of an administration of open-label placebo pills including the instruction to take two pills a day for six weeks. The pill intake in both OLP intervention groups starts at the very day after the study visit (t0). The study visit (t0) occurs during the first 14 days of the menstrual cycle of each participant, i.e., in the follicular phase. Due to the cyclicity of PMS symptoms, we decided to provide the intervention for a longer period of time in contrast to previous OLP studies [1-3] to encompass at least one whole menstrual cycle. After the explicit mentioning of the pharmacological inertness of the pills in both groups, the OLP+ group receives - in contrast to the OLP- group - additionally a comprehensive OLP treatment rationale (see supplementary material 3) at t0 as well as at t1. The discussion points encompass: a) that placebos can be powerful, also for PMS; b) an explanation on why placebos work; c) that faithful pill intake is crucial and that doubts do not have a negative effect on the treatment; d) that placebos have also been shown to work without deception. Moreover, a video sequence of about 2 minutes is shown of an American NBC news report with German subtitle about an OLP intervention on irritable bowel syndrome including a patient report (see: https://www.youtube.com/watch?v=uv0SuWKZjsI&t=1s). Neither participants nor study team members are blinded. Yet besides the treatment provider, study team members are not informed about group allocation to minimise investigator bias. As investigational product, pink oral placebo pills - “P-Dragees rosa Lichtenstein” of the German brand *Winthrop* - are administered, which are certified placebo pills without any active ingredient

and with validated quality. Each pill contains the following substances: lactose monohydrate; magnesium stearate (Ph. Eur.); microcrystalline cellulose; highly dispersed silicon dioxide; white clay, magrogol glycerolhydroxy stearate (Ph. Eur.); Arabic gum; montanglycol wax; povidone (K 25); talcum; titanium dioxide (E 171); erythrosine; aluminium salt (E 127); calcium carbonate; sucrose; glucose syrup; maize starch; macrogol 6000. Also, participants of both intervention groups receive a certificate, confirming that the study medication only contains placebo pills.

3.) The OLP- group obtains the same intervention and intake instruction like the OLP+ group and the proceeding is identically. However, no treatment rationale or further treatment explanation is provided.

Detailed screening and PMS assessment

After informed consent obtainment, participants are comprehensively screened for study eligibility. Therefore, the following information is collected online:

- Verification of eligibility according to eligibility criteria
- Medical and gynaecological history
- Assessment of sociodemographic data
- Assessment of PMS retrospectively

In a second step, women fulfilling eligibility criteria and PMS criteria retrospectively, start a prospective PMS screening with daily online surveys as part of a PMS symptom diary assessment for one menstrual cycle for the screening and in total for three menstrual cycle for primary outcome assessment (see below).

For peer review only

OUTCOME MEASURES: PRIMARY, SECONDARY, AND TERTIARY

Primary outcome measures

The German PMS symptom diary consists of 27 symptoms and of three further items assessing interference in different domains (i.e., work or school; social activities; relations with others). Usually, a four point Likert scale is employed, however, in line with Endicott et al. [17] we apply a six point Likert scale to be able to detect subtle changes. We compare changes over time of PMS symptom intensity and interference across three menstrual cycles. The *PMS symptom intensity score* is derived from the first 27 items of the German PMS symptom diary, and the *symptom interference score* is derived from the last three items. With regard to symptom intensity, two additional subscales are calculated by dividing the 27 symptom items into a psychological symptom intensity subscale and a physical symptom intensity subscale score (see secondary outcomes) [17].

Following Kues et al. [18] and Janda et al. [16], we calculate separate scores for the luteal and follicular phase and consider the difference for our analysis. In more detail, we conservatively consider 7 days prior to menstruation onset as a timeslot for the luteal phase and 7 days of the follicular phase, whereas the start of the follicular phase for each participant is calculated using the following formula: (individual cycle duration in days-14)/2 = first day of the follicular phase. Subsequently, we calculate the difference score of the means for each menstrual cycle for the respective primary outcome of interest (i.e., for PMS symptom intensity or interference). Intensity and interference are considered as marked if rated by participants with at least a 4 (very strong) on a scale from 0 (not at all) to 5 (extreme). Moreover, the item has to be rated with at least a four for at least two consecutive days to be considered as marked. Further, symptoms only are considered as marked symptoms if the respective symptom is not marked in the follicular phase and if it is associated with

For peer review only - http://bmjopen.bmj.com/site/about/guidelines.xhtml

marked interference in the luteal phase. If a participant has four or more symptoms marked in the luteal phase, also interference of only one day is accepted. We are also interested in the trajectory of the symptoms in the intervention groups across the two luteal phases after t0. Hence, we will compare if there are differences of group effects at cycle 2 (C2) and cycle 3 (C3) in contrast to cycle 1 (C1).

Moreover, we evaluate the effect of a comprehensive OLP treatment rationale, by providing one intervention group with a comprehensive treatment rationale in addition to intake instructions whereas the other intervention group only obtains intake instructions (see also *Trial treatment and study arms*).

Secondary outcome measures

Secondary outcomes comprise the following:

- Physical and psychological symptom subscale of the Ger*man PMS Symptom Diary* [16]*:*

  To test whether an OLP response occurs primarily on physical or on psychological symptoms, the above-mentioned German PMS Symptom Diary is used including the aforementioned calculation methods. The following scores are derived from the diary data of three menstrual cycles:

  - Physical symptom subscore: 14 items of the German PMS symptom diary are considered: depressed mood; hopelessness; feeling of worthlessness; feeling anxious; feeling tensed or being stressed out; irritability; sudden attacks of sadness; sudden crying; sensitivity to rejections; feeling angry; controversies with others; loss of interest in common activities; impaired concentration; feeling out of control or overwhelmed.

For peer review only - http://bmjopen.bmj.com/site/about/guidelines.xhtml

  - Psychological symptom subscore: The following seven items are considered: Breast tenderness; breast sensitivity; breast swelling; headache; joint pains; muscle pain; feeling of a general body swelling; weight gain.

- *PMS-Impact Questionnaire* (PMS-I) [19]:

  The PMS-I measures the impact of premenstrual syndromes with 18 items and provides a total score and two subscales - a psychological impact and a functional impact scale. It was found to be a valid, reliable (Cronbach's alpha = .90) and economic assessment tool of PMS impact [16]. The questionnaire differentiates between functional and psychological impact [19]. In comparison to the German PMS symptom diary, it covers more domains of daily life (e.g., sexual experiences, physical exertion, stress coping) and is more specific for the detection of small changes of PMS impact. The PMS-I is assessed three times during the luteal phase, once during the prospective screening (L1) and twice during the intervention phase (L2 & L3).

- *Short Form-12 Health Survey for health-related quality of life* (SF-12) [20]:

  The SF-12 measures health-related quality of life independent of disorders, comprising 12 items, providing a physical and mental component summary scale. Health-related quality of life is assessed since PMS symptoms have a high impact on women's quality of life [21]. Physical health is comprised of the dimensions of physical functioning, physical role-functioning, bodily pain, general health; mental health is comprised of the dimensions of vitality, social functioning, emotional role-functioning, and mental health. The SF-12 is a validated and economic version of the SF36. For our purposes, we ask for ratings of the last week including today (premenstrual phase). The SF-12 is

For peer review only - http://bmjopen.bmj.com/site/about/guidelines.xhtml

completed three times by participants: during the luteal phase of the prospective screening (L1) and twice during the intervention phase (L2 & L3).

- *Hospital Anxiety and Depression Scale – German Version* (HADS-D) [22]:
  The HADS-D aims to assess anxiety and depression in individuals with somatic conditions and can be used for self-reported severity evaluation. It comprises two subscales (anxiety and depression) with seven items each and with a four-level answer format. The sum score indicates general psychological interference. We decided to use the HADS-D since it is a very sensitive tool for less severe cases of psychological disorders, which is usually the case for patients in a primarily somatic setting. We assess the HADS-D only at L1 during the first luteal phase before the start of the intervention.
- *A scale for assessment of satisfaction in close relationships* (ZIP) [23]:
  The ZIP is the German version of the relationship assessment scale by Hendrick et al. [24] and measures satisfaction in romantic relationships. The questionnaire entails seven items and had been shown to have high reliability and a clear unifactorial structure [24]. The ZIP is rated three times by participants during the luteal phases of the prospective screening (L1) and twice during the intervention phase (L2 & L3).
- Treatment adherence is measured by counting the remaining pills:
  Participants are asked to return the blister and pill boxes after the intervention.
- Expectancy of relief and desire for relief of premenstrual symptom severity are assessed at t0 by means of a Likert scale ranging from 1-6, like the primary outcome measure (German PMS Symptom Diary) and three weeks after intervention start.
- *Complementary and Alternative Medicine Beliefs Inventory* (CAMBI) [25]:

For peer review only

The CAMBI serves to assess three different aspects of complementary alternative medicine intervention beliefs with a 17-item questionnaire with satisfactory validity and reliability measures. Three dimensions comprise beliefs in natural treatments, participation in treatment, and holistic health. Participants fill out the inventory posttreatment at t2.

- Item about placebo appraisal of the *Questionnaire on Responders' Attitude Regarding Non-Specific Therapies* [26]:
  At t2, participants rate the item about placebo appraisal online which encompasses the following three questions:
  1) How would you define the term placebo?
  2) A rating of the positivity or negativity of the term.
  3) Do you believe that physical complaints can get better if you merely belief in the efficiency of a therapy?
- Patient-provider connection items of the *Healing Encounters and Attitudes Lists – German Short Version* (HEAL-D-SF) [27]: The HEAL-S provides an item bank to measure nonspecific factors in treatment. We collect the patient-provider connection item at t2 to control for the influence of provider perception.
- Placebo intervention credibility is assessed online at t2 with the question:
  Did you believe that you received placebo pills during the study, i.e. pills without any pharmacological content? The three answer options are:
  1) Yes, I was sure that I received placebo pills.
  2) I had doubts that I received placebo pills
  3) No, I did not believe that I received placebo pills.
  Moreover, the OLP- group is asked whether they felt something was missing, whereas the OLP+ group is asked whether they found the provided rationale convincing and how it may have helped or confused them personally.

For peer review only - http://bmjopen.bmj.com/site/about/guidelines.xhtml

Tertiary outcomes measures

Other, tertiary outcomes of interest are:

- Sociodemographic variables: age, family and relationship status, sexual orientation, citizenship, country of birth, mother tongue, educational degree, employment status, height, weight, and number of births and aborts, if participant has a GP/gynaecologist.
- Retrospective PMS Questionnaire sum score
- Mini-International Neuropsychiatric Interview (MINI)
- Pregnancy test
- Medical and gynaecological history
- Concomitant pharmacological or non-pharmacological interventions and changes in all groups
- Qualitative answers regarding treatment and study experience
- Occurrence of side-effects/adverse events
- Number of participants who withdraw from study participation
- Number of participants from the TAU group who attend an open-label placebo intervention after study conclusion
- Number of participants from the intervention groups who show interest to continue the open-label placebo intervention after study conclusion
- Suicidality

Adverse events and safety monitoring

Study participants will be asked about any side-effects, including also treatment-emergent adverse-events (TEAEs) or serious adverse events (SAEs) at t1 and t2. Suicidality, which is specifically associated with premenstrual dysphoric disorders [28], and generally found to be higher in the luteal (=premenstrual) phase [29], will also be assessed at each study visit and if necessary, safety actions will be provided.

For peer review only - http://bmjopen.bmj.com/site/about/guidelines.xhtml

SAMPLE SIZE AND STATISTICAL ANALYSIS OF DATA

Data management

For data management, Microsoft Excel® and LabKey®, which is hosted by the University of Basel, is used. Study data is collected at the web server-based software LimeSurvey®. Only authorized study team members are able to view and export data. Entries and actions are marked with the initials of the respective study member.

Sample size

A calculation with a power analysis on the basis of an F-test and an ANCOVA for three groups, using the statistical software G*Power, indicated a total sample size of 206 participants needed for a power of 0.9 and a total sample size of 158 participants for a power of 0.8 to detect a medium effect size of f=0.25 (equals d=0.5) with an alpha-level of 0.05.

Since we will adopt a multilevel approach which enables more precise estimations, we also conducted a power analysis on a repeated-measures ANOVA, using G*Power, whereby we are interested in the interaction between time (menstrual cycles, 3 levels) and study groups (3 levels). If the correlation for the outcome among the different time points (1-3 cycles) is assumed to be 0.5, then based on an alpha-level of 0.05, a power of 0.8, and a medium effect size of f=0.25, the required samples size is 36 (12 per group). Accordingly, for the main effect of menstrual cycle, we would need 30 participants (10 per group), and for the main effect of study group we would need 108 participants (36 per group). On this basis, we chose a conservative total sample size of 150 participants (50 per group). It is worth noting that most open-label placebo studies reported effect sizes that are generally higher than d=.5 (e.g., chronic low back pain: d=.77 [1]; irritable bowel syndrome: d=.79 [2]). However, in another open-label placebo study for patients suffering from major

For peer review only - http://bmjopen.bmj.com/site/about/guidelines.xhtml

depressive disorder [5], the authors reported an effect size of d=.54 whilst stating that the study was limited by low statistical power (i.e., the findings did not support the hypothesis that open-label placebos are effective in depression). Since PMS is not only characterised by physical complaints, yet also by psychological symptoms such as depressive mood, and since there is no specific research on open-label placebo effects and effect sizes in PMS, we considered a conservative medium effect size as more appropriate. This resulted in a more conservative sample size.

Statistical analysis

For the testing of the primary hypotheses, the daily data from three menstrual cycles of a symptom diary is examined. Because of the hierarchical structure of the data (menstrual cycles as level-1 are nested within participants as level-2) and given our aim to predict post-interventional changes between menstrual cycles as the primary outcome, a multilevel modelling approach will be adopted. Multilevel models take care of the interdependence of the hierarchical data that arises due to the fact that observations within the same individual are typically more similar to each other compared to observations between individuals [30 31]. Here, we will analyse temporal changes across menstrual cycles of symptom intensity and interference among the different study groups. To compare a TAU group with two intervention groups and to evaluate the effect of the applied treatment rationale, the following linear *a priori* contrast will be tested for the factor study group: TAU < OLP- < OLP+. Thus, based on published findings [6], we expect that the effect of OLP- lies between that of TAU and OLP+. Out statistical model contains menstrual cycle as within-subject factor with three levels (C1, C2, and C3), study group as between-subject factor with three levels (TAU, OLP+, and OLP–), and the interaction between the two factors. In line with recommendations by Fitzmaurice et al.[32], we will treat baseline values (i.e.,

For peer review only - http://bmjopen.bmj.com/site/about/guidelines.xhtml

values at C1) as a part of the outcome vector and assume that the means among study groups are equal at baseline since participants are randomized. The model contains the two main effects study group contrast (defined above) and menstrual cycle (temporal effect) plus the interaction between the two. We will be primarily interested in the interaction term as it tests for the differential temporal change between study groups in order as defined in the linear contrast.

Analyses of secondary outcomes are based on the same multilevel model as used for primary outcomes, but will be considered exploratively, thereby not correcting for multiple tests [33].

Monitoring

The study is monitored for quality and regulatory adherence. The monitor, who is not involved in our study and approved by the local ethical committee, verifies the qualification of the investigators and study team members, and monitors sound and appropriate documentation.

Ethics and dissemination

The present protocol and applied informed consent forms were approved with regard to their content and compliance with ethical regulations by the *Ethics Committee for Northwest/Central Switzerland*. The study is carried out with principles enunciated in the current version of the Declaration of Helsinki [34] and the guidelines of Good Clinical Practice (GCP) issued by ICH [35].

Women interested in study participation receive a participant information sheet and consent form, describing the study and providing sufficient information for an informed decision about participation and data confidentiality and detailed oral information is provided. The results of the planned analyses will be published in a peer-reviewed journal.

For peer review only - http://bmjopen.bmj.com/site/about/guidelines.xhtml

DISCUSSION

Given the huge burden of women suffering from PMS [11 36], it is necessary to optimize treatment options and to enhance the understanding of intra-interventional processes in PMS treatment. Moreover, interventions - including medical or psychotherapeutic treatment - can generally benefit from an increased comprehension of treatment context-factors and related meaning responses [37 38] involved in ailment amelioration. We therefore address the investigation of PMS-interventional context-factors and OLP effects and mechanisms with our study and strive to contribute to further knowledge in both areas. Previous RCTs of PMS interventions indicate high placebo responses [7]. The presented trial is essential to test whether an OLP response can also be elicited in PMS and whether an amelioration of symptoms occurs primarily on a somatic or psychological level. Additionally, we assess the importance of a comprehensive treatment rationale in a clinical population independent of a specific intervention.

So far there have been different aspects of previous OLP studies which have been critically evaluated in a meta-analysis of clinical OLP studies [39]. For instance, a lack of an adequate control group in OLP trials has been pointed out. To address this issue, the TAU control group of our trial has the same amount of contact with the study staff as the two intervention groups. Furthermore, we added an OLP- group to test the effect of a comprehensive OLP treatment rationale in a clinical population. Hence, we aim to assess whether placebo pills alone are sufficient or whether a comprehensive treatment rationale is vital to elicit an OLP response and, thus, strive to distinguish OLP effects from positive framing [39].

Moreover, to tackle previous critique of potential researcher bias and lack of blinding [39], any interactions with participants regarding the study proceedings, including data collection, are conducted by study team members who do not provide the

For peer review only - http://bmjopen.bmj.com/site/about/guidelines.xhtml

intervention and who are not informed about study group allocation at the study visit. The intervention is provided by one person only (A.F.N.) who is not involved in any interactions related to data collection. Furthermore, we decided to not provide the treatment rationale to all participants, primarily to test the effect of the treatment rationale and to avoid any experiences of disappointment in the TAU control group. A further advantage of our study is the large planned sample of 150 participants, tackling a further previously mentioned critique point [39].

Because we are interested in understanding mechanisms of OLP responses, we assess an array of different questionnaires including attitudes and experiences of participation in the different study groups. In this sense, we aim to address different aspects of former criticism with our study design and to enhance knowledge about OLP effects.

To sum up, a positive OLP response in PMS would form the basis for future studies investing how to successfully harness placebo effects, in an ethical fashion, in clinical practice and to optimize PMS interventions that often entail side-effects.

For peer review only

AUTHOR CONTRIBUTIONS

All of the following authors contributed significantly to the conception of the presented study and to the manuscript: Antje Frey Nascimento (email: antje freynascimento@unibas.ch), Jens Gaab (email: jens.gaab@unibas.ch), Irving Kirsch (email: irvkirsch@gmx.de), Joe Kossowsky (email: joe.kossowsky@unibas.ch), Andrea Meyer (email: andrea.meyer@unibas.ch), Cosima Locher (email: cosima.locher@unibas.ch)

FUNDING

This study is supported by the Swiss National Science Foundation (Prof. Dr. Jens Gaab), grant number: 325130_170117, under the name “Taking the placebo further: open placebo”. Cosima Locher, PhD, received funding for this project from the Swiss National Science Foundation (SNSF): P400PS_180730 (Title: Overcoming Classificatory and Methodological Hurdles to Improve Treatment of Chronic Primary Pain: A Network Meta-Analytic Approach)

COMPETING INTERESTS

There are no known potential conflicts of interest.

ETHICAL APPROVAL

This study has been approved by the EKNZ Ethical Committee (ID 2017-02186; Open-label placebo treatment of women with premenstrual syndrome: A randomized controlled trial)

WORD COUNT

The manuscript entails 4949 words.

For peer review only - http://bmjopen.bmj.com/site/about/guidelines.xhtml

REFERENCES

1. Carvalho C, Caetano JM, Cunha L, et al. Open-label placebo treatment in chronic low back pain: a randomized controlled trial. *Pain* 2016;157(12):2766.
2. Kaptchuk TJ, Friedlander E, Kelley JM, et al. Placebos without deception: a randomized controlled trial in irritable bowel syndrome. *PLoS One* 2010;5(12):e15591. doi: 10.1371/journal.pone.0015591
3. Schaefer M, Harke R, Denke C. Open-Label Placebos Improve Symptoms in Allergic Rhinitis: A Randomized Controlled Trial. *Psychother Psychosom* 2016;85(6):373-74. doi: 10.1159/000447242
4. Hoenemeyer TW, Kaptchuk TJ, Mehta TS, et al. Open-label placebo treatment for cancer-related fatigue: a randomized-controlled clinical trial. *Scientific reports* 2018;8(1):2784.
5. Kelley JM, Kaptchuk TJ, Cusin C, et al. Open-label placebo for major depressive disorder: a pilot randomized controlled trial. *Psychother Psychosom* 2012;81(5):312-4. doi: 10.1159/000337053
6. Locher C, Nascimento AF, Kirsch I, et al. Is the rationale more important than deception? A randomized controlled trial of openlabel placebo analgesia. *Pain* 2017
7. Green L, O’Brien P, Panay N, et al. Management of Premenstrual Syndrome: Green-top Guideline No. 48. *BJOG* 2017;124(3):e73-e105. doi: 10.1111/1471-0528.14260
8. Van Ree JM, Schagen Van Leeuwen JH, Koppeschaar HP, et al. Unexpected placebo response in premenstrual dysphoric disorder: implication of endogenous opioids. *Psychopharmacology (Berl)* 2005;182(2):318-9. doi: 10.1007/s00213-005-0090-8
9. Sampson GA. Premenstrual syndrome: a double-blind controlled trial of progesterone and placebo. *The British Journal of Psychiatry* 1979;135(3):209-15.
10. Yonkers KA, O'Brien PMS, Eriksson E. Premenstrual syndrome. *The Lancet* 2008;371(9619):1200-10. doi: 10.1016/s0140-6736(08)60527-9
11. Direkvand-Moghadam A, Sayehmiri K, Delpisheh A, et al. Epidemiology of Premenstrual Syndrome (PMS)-A systematic review and meta-analysis study. *Journal of clinical and diagnostic research: JCDR* 2014;8(2):106.
12. Maharaj S, Trevino K. A Comprehensive Review of Treatment Options for Premenstrual Syndrome and Premenstrual Dysphoric Disorder. *J Psychiatr Pract* 2015;21(5):334-50. doi: 10.1097/PRA.0000000000000099
13. O'Brien PS, Rapkin A, Schmidt PJ. The premenstrual syndromes: PMS and PMDD: CRC Press 2007.
14. Freeman EW, Rickels K. Characteristics of placebo responses in medical treatment of premenstrual syndrome. *American Journal of Psychiatry* 1999;156(9):1403-08.
15. Kaptchuk TJ, Shaw J, Kerr CE, et al. “Maybe I made up the whole thing”: placebos and patients’ experiences in a randomized controlled trial. *Culture, Medicine, and Psychiatry* 2009;33(3):382-411.
16. Janda CP, Kues JNP, Andersson GP, et al. A symptom diary to assess severe premenstrual syndrome and premenstrual dysphoric disorder. *Women Health* 2016:1-18. doi: 10.1080/03630242.2016.1206055
17. Endicott J, Nee J, Harrison W. Daily Record of Severity of Problems (DRSP): reliability and validity. *Arch Womens Ment Health* 2006;9(1):41-9. doi: 10.1007/s00737-005-0103-y
18. Kues JN, Janda C, Kleinstäuber M, et al. Internet-based cognitive behavioural self-help for premenstrual syndrome: study protocol for a randomised controlled trial. *Trials* 2014;15(1):472.

For peer review only - http://bmjopen.bmj.com/site/about/guidelines.xhtml

19. Kues JN, Janda C, Kleinstauber M, et al. How to measure the impact of premenstrual symptoms? Development and validation of the German PMS-Impact Questionnaire. *Women Health* 2016;56(7):807-26. doi: 10.1080/03630242.2015.1118734
20. Ware Jr JE, Kosinski M, Keller SD. A 12-Item Short-Form Health Survey: construction of scales and preliminary tests of reliability and validity. *Medical care* 1996;34(3):220-33.
21. Nevatte T, O'Brien PM, Backstrom T, et al. ISPMD consensus on the management of premenstrual disorders. *Arch Womens Ment Health* 2013;16(4):279-91. doi: 10.1007/s00737-013-0346-y
22. Herrmann C, Buss U, Snaith R. HADS-D: hospital anxiety and depression scale-German version. *Bern: Hans Huber* 1995;1:995.
23. Hassebrauck M. ZIP–Ein Instrumentarium zur Erfassung der Zufriedenheit in Paarbeziehungen. *Zeitschrift für Sozialpsychologie* 1991;22(4):256-59.
24. Hendrick SS, Dicke A, Hendrick C. The relationship assessment scale. *Journal of Social and Personal Relationships* 1998;15(1):137-42.
25. Bishop FL, Yardley L, Lewith G. Developing a measure of treatment beliefs: the complementary and alternative medicine beliefs inventory. *Complement Ther Med* 2005;13(2):144-9. doi: 10.1016/j.ctim.2005.01.005
26. Faessler M, Gnadinger M, Rosemann T, et al. Placebo interventions in practice: a questionnaire survey on the attitudes of patients and physicians. *Br J Gen Pract* 2011;61(583):101-7. doi: 10.3399/bjgp11X556209
27. Greco CM, Yu L, Johnston KL, et al. Measuring nonspecific factors in treatment: item banks that assess the healthcare experience and attitudes from the patient's perspective. *Quality of Life Research* 2016;25(7):1625-34.
28. Pilver CE, Libby DJ, Hoff RA. Premenstrual dysphoric disorder as a correlate of suicidal ideation, plans, and attempts among a nationally representative sample. *Soc Psychiatry Psychiatr Epidemiol* 2013;48(3):437-46. doi: 10.1007/s00127-012-0548-z
29. Saunders KE, Hawton K. Suicidal behaviour and the menstrual cycle. *Psychol Med* 2006;36(7):901-12. doi: 10.1017/S0033291706007392
30. Kalmbach DA, Kingsberg SA, Ciesla JA. How changes in depression and anxiety symptoms correspond to variations in female sexual response in a nonclinical sample of young women: a daily diary study. *The journal of sexual medicine* 2014;11(12):2915-27.
31. Field A, Miles J, Field Z. Discovering statistics using R: Sage publications 2012.
32. Fitzmaurice GM, Laird NM, Ware JH. Applied longitudinal analysis: John Wiley & Sons 2012.
33. Streiner DL, Norman GR. Correction for multiple testing: is there a resolution? *Chest* 2011;140(1):16-18.
34. WMA. World Medical Association Declaration of Helsinki: Ethical Principles for Medical Research Involving Human Subjects. *JAMA* 2013;310(20):2191-4. doi: doi: 10.1001/jama.2013.281053
35. ICH. International Conference on Harmonization-E6 Guideline for Good Clinical Practice 1996 [Available from: http://www.ich.org/fileadmin/Public_Web_Site/ICH_Products/Guidelines/Efficacy/E6/E6_R2__Step_4.pdf.
36. Halbreich U, Borenstein J, Pearlstein T, et al. The prevalence, impairment, impact, and burden of premenstrual dysphoric disorder (PMS/PMDD). *Psychoneuroendocrinology* 2003;28:1-23. doi: 10.1016/s0306-4530(03)00098-2

For peer review only - http://bmjopen.bmj.com/site/about/guidelines.xhtml

37. Moerman DE, Jonas WB. Deconstructing the placebo effect and finding the meaning response. *Annals of Internal medicine* 2002;136(6):471-76.
38. Annoni M, Blease C. A Critical (and Cautiously Optimistic) Appraisal of Moerman's "Meaning Response". *Perspect Biol Med* 2018;61(3):379-87. doi: 10.1353/pbm.2018.0050
39. Charlesworth JEG, Petkovic G, Kelley JM, et al. Effects of placebos without deception compared with no treatment: A systematic review and meta-analysis. *J Evid Based Med* 2017;10(2):97-107. doi: 10.1111/jebm.12251

For peer review only

FIGURE LEGENDS

Figure 1. *Study Design and Flow of Participants*

Figure 2. *Overview of Study Groups*

SUPPLEMENTARY MATERIAL

1) Study information and informed consent form (English)

2) Study information and informed consent form (original; in German)

3) Oral information of the open-label placebo treatment rationale

For peer review only

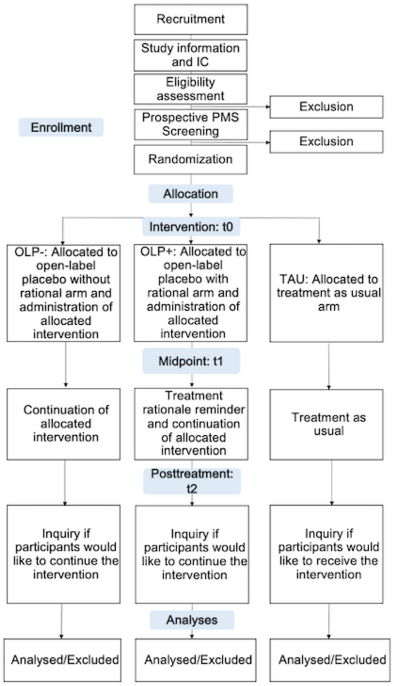

Figure 1. Study Design and Flow of Participants

90x90mm (300 x 300 DPI)

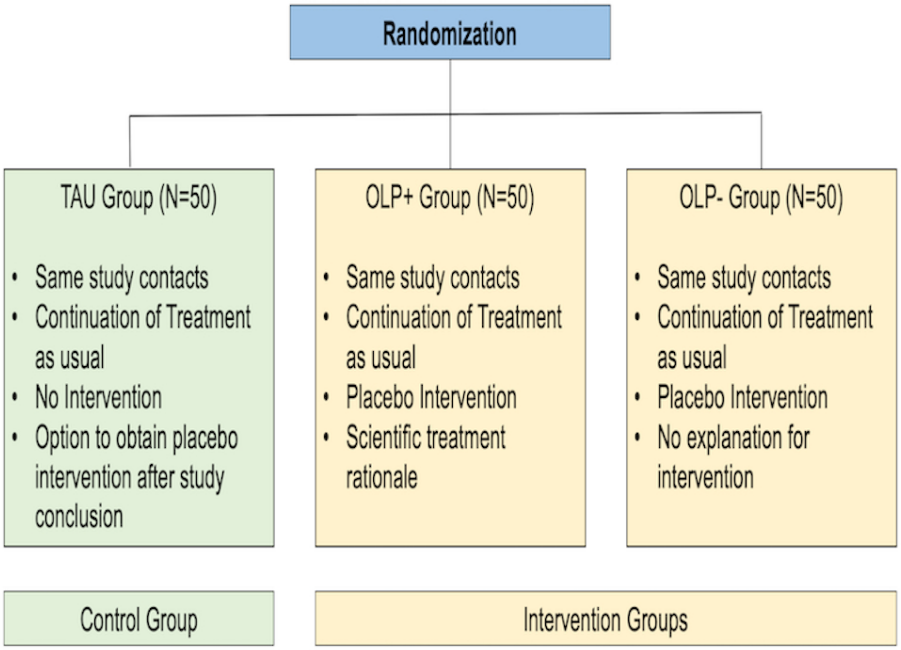

Figure 2. Overview of Study Groups

90x90mm (300 x 300 DPI)

SUPPLEMANTARY MATERIAL

Table of Content

1) Study information and informed consent form (English)……………………………………2

2) Study information and informed consent form (original; in German)..…………………...10

3) Oral information of the open-label placebo treatment rationale…………………………..18

For peer review only

For peer review only - http://bmjopen.bmj.com/site/about/guidelines.xhtml

1) STUDY INFORMATION AND INFORMED CONSENT FORM (ENGLISH)

Efficacy study of an integrative and side effect free intervention method of premenstrual complaints

This study is organized by:

University of Basel
Faculty of Psychology
Division of Clinical Psychology and Psychotherapy
Missionsstrasse 62a
4055 Basel, Switzerland

Dear Madame,

We would like to ask if you want to participate in a clinical efficacy study. In the following, our study proceeding will be delineated:

1. Aim of the study
We test a novel, integrative and side effect free treatment method of premenstrual complaints, which already showed good efficacy for irritable bowel syndrome and chronic low-back pain. In this context, placebo pills will also be administered.

2. Selection
Inclusion:
All women who suffer from a moderate to severe premenstrual syndrome (in the following called PMS) and who are between 18 and 45 years of age can participate. Further, participants should have a regular menstrual cycle. PMS severity should warrant the women's wish for a treatment and should interfere with daily life (e.g., indicated by missing at work, conflicts with close people).

Exclusion criteria:
- You are pregnant or breastfeeding
- You suffer from a severe psychological or somatic disease
- You take drugs or consume a large amount of alcohol or you take other psychoactive substances
- You have been starting the intake of a new medication within the last 30 days
- You are sterilized, your uterus or appendices were (partly) removed
- You have a body mass index of 30 or higher
- You are currently in psychotherapeutic treatment due to premenstrual complaints
- You react allergic to lactose, magnesium, or other contents of the placebo pills (pharmacological harmless and inactive substances)

3. General information
With this national study, a novel and promising treatment method of women with PMS is tested. In this context, placebo dragées will also be administered, i.e., dragées without any pharmacological content. The study entails a screening phase of approximately one month and an intervention phase of approximately two months. Study participants will fill out daily symptom diaries for approximately 3 months (approx. 2 min./day).
There will be one ambulant study visit at the University of Basel (Missionsstrasse 62a, 4055 Basel) during the intervention phase, which will take about 60 minutes. Additionally, there will be two conversations on the phone and online surveys. We plan to include 150 participants in the study. The intake of any hormonal or other complain-specific medication can be continued.

For peer review only - http://bmjopen.bmj.com/site/about/guidelines.xhtml

This study complies with Swiss laws. Furthermore, we consider all international acknowledged guidelines. The responsible cantonal ethical committee already proved and accepted this study. A description of this study can also be found on the homepage of the federal office of health: www.kofam.ch (registration number: SNCTP000002809*).*

4. Proceeding

There are three study groups and you will be allocated to one of them by chance: A control group, a placebo group, and a treatment group. Participants allocated to the control group have the opportunity to obtain the novel treatment approach after study conclusion. Participants of the placebo group receive a placebo treatment in an open manner (i.e., they will be told that they receive a placebo). Participants of the treatment group receive an integrative and side effect free treatment method, which also comprises an open administration of placebos.

- Independent from the study group, we will ask you to conclude PMS diary questions daily online (for about three months).
- Additionally, during the screening phase, you will be asked to conclude the first online survey. Moreover, we will conduct further online surveys during the premenstrual phases, i.e., across three months at three time points. In addition, there will be one last online assessment at the end of the study after the conclusion of the treatment.
- At the beginning of the intervention phase, there will be one study visit in our division during which we will conduct the intervention depending on study group allocation. In advance, we conduct a screening of psychological disorders as well as a pregnancy test. Subsequently, in the placebo group and the treatment group the placebo dragées will be administered. After approximately one month there will be a midpoint contact – on the phone or if desired in person. After study conclusion we will contact you again on the phone.
- If you have been taking already any medication for at least 30 days against PMS complaints you can continue to take them.
- The whole study lasts about approximately three months. You will need around two minutes for the conclusion of the online PMS symptom diary each day. The study visit in our division will take about 60 minutes, the midpoint contact approximately 15 minutes, and the last contact about 10 minutes. The five additional online surveys will take about 10 minutes on average. Table 1 shows an overview of the planned contacts and online surveys and the estimated durations.

Table1. Study proceeding

**STUDY**	**When?**	**Where?**	**Content?**	**Duration?**
SCREENING PHASE				
Oral/written study information	At the very beginning	On phone/ mail	You will be informed about the study in detail and about your rights	Approx. 10 minutes
First screening survey	Directly after the first phone contact	Online	Clarification of eligibility criteria<br><br>Assessment of personal data<br><br>Brief PMS screening	Approx. 20 minutes
PMS diary screening	During three menstrual cycle	Online	You will be asked once a day to rate your symptom intensity and interference of daily life	Daily approx. 2 minutes for at least one menstrual cycle

For peer review only - http://bmjopen.bmj.com/site/about/guidelines.xhtml

PMS survey	1. Premenstrual phase	Online	Questionnaires	Approx. 10 minutes
Information about study participation	Directly after onset of menstruation, during second menstrual cycle	Call	Fulfillment of eligibility criteria, appointment of the study visit	Approx. 5 minutes
INTERVENTION PHASE				
Personal study visit	During the first 14 days of the second menstrual cycle, after screening	At the University Basel	Last screening of psychological disorders and pregnancy test as exclusion criteria<br><br>Group-specific intervention<br><br>Collection of online questionnaires after intervention	Approx. 60 minutes
PMS survey	2. Premenstrual phase	Online	Questionnaires	Approx. 10 minutes
Midpoint contact	Approx. one month after study visit	Phone/ Skype or personally	Group-specific intervention	Approx. 15 minutes
PMS survey	3. Premenstrual phase	Online	Questionnaires	Approx. 10 minutes
Post-treatment contact	After approx. three months in total	Phone	Closure	Approx. 10 minutes
Post-treatment survey	After post-treatment contact	Online	Closure questionnaires	Approx. 20 minutes
Returning of the pill packages (possibly with remaining pills) in treatment group				
Money transfer of reimbursement				

For peer review only

For peer review only - http://bmjopen.bmj.com/site/about/guidelines.xhtml

5. Benefit

With the participation in our study, you have the opportunity to take part in a novel, integrative, and side effect free intervention. This will support research of side effect free treatment methods. The conclusion of a symptom diary by itself can help to improve PMS symptom management. Additionally, results of this study can be important for a further understanding of PMS interventions, of common factors of the therapeutic context as well as for the ethical implementation of placebos in clinical research.

6. Rights

Your participation is voluntary. If you do not participate or if you will withdraw your participation later, you do not have to give any explanation. If you stop study participation, there will not be any negative consequences. You can ask questions regarding study participation at any point. If you have questions, please contact the person mentioned at the end of this information.

7. Duties

For participants it is also necessary to adhere to the necessary demands and requirements. This includes compliance with arranged appointments and the prompt conclusion of online surveys. In addition, we ask you to inform the investigator of the trajectory of your symptoms and to report new symptoms, essential increase of symptoms, or other relevant changes of your well-being. We ask you to inform the investigator also about concomitant interventions and therapies of your physician and of the intake of any current medication as well as changes in medication doses – also during the course of the study.

8. Risks and exposures for participants

There are no specific risks to be expected due to study participation. The occupation with your PMS complaints could lead to an enhanced perception of your symptoms, but also to an ameliorated symptom management which in turn could lead to a decrease of your complaints.

For women who could get pregnant

If you become pregnant during study participation please directly inform us. The study participation will then prematurely be finished since we are not able to use your data.

9. Other options of treatment

There are tested treatment possibilities for interfering premenstrual complaints about which your physician or gynecologist can inform you. Of course, this is also still possible after study participation.

10. Results of the study

The investigator informs study participants during the study about any new findings which could influence the benefit of this study and therefore influence also your consent of study participation. We will inform you about incidental findings which could be helpful for the diagnosis and treatment of any present disorder. If you do not want to be informed, please inform the investigator ahead of study participation.

11. Confidentiality of data and samples

For this study, your personal and medical data will be recorded. For study purposes, data will be coded during data collection. Coded means that all referred data, which could identify who you are (name, date of birth) will be eliminated and be replaced by a key. All who do not know the key cannot deduce who you are. The list of keys will at all times remain at the institution (faculty of psychology). All persons who can access your data underlie the duty of confidentiality. All

For peer review only - http://bmjopen.bmj.com/site/about/guidelines.xhtml

requirements of confidentiality will be respected and we will not mention your name in any publication or on the internet. As a participant you have at all times the right to access your data.

It is possible that this study will be reviewed by the responsible ethics committee or by the University of Basel. The investigator will possibly have to disclose your personal or medical data in the context of external reviewing.

12. Withdrawal
You can withdrawal from study participation at any times if you wish to. Your coded data will be used until the point of withdrawal, because if not the project will lose quality. After data analysis your data will be completely anonymized after withdrawal, i.e., the key allocation will be erased so that as a consequence nobody can find out that the data stems from you originally.

13. Reimbursement of participants
If you participate in this study and complete in full all steps you will receive 200 CHF as reimbursement or 25 signatures (for psychology students). 70 CHF will be reimbursed for the full completion of the screening phase and further 130 CHF for participation of the complete intervention phase, including the completion of all online surveys. Expenses such as loss of working hours or travel expenses which are due to study participation are already included in the mentioned reimbursement. For you or your health insurance no costs will accrued due to study participation.

14. Liability
The institution which arranges this study and which is responsible for the study conduct is liable in case of any harm, which could be caused in relation to tested substances or any research activities (e.g., examinations). The prerequisites and proceeding therefor are regulated by law. In case that you suffer any damage, please consult the investigator.

15. Funding of the study
The study is funded by the *Swiss National Science Foundation*.

16. Contact persons
At any moment you can contact one of the following contacts if you have any questions, uncertainties, or emergencies during or after study participation:

Antje Frey Nascimento M Sc
Missionsstrasse 60/62
Phone: (+41) 061 207 05 98
antje.freynascimento@unibas.ch

Dr. phil. Cosima Locher
Missionsstrasse 60/62
Phone: (+41) 061 207 03 85
cosima.locher@unibas.ch

Study email address: pms-studie-psychologie@unibas.ch

17. Glossary of terms in need of explanation:

- What means “placebo"?
  A placebo looks like a real medication and also is wrapped like real medication. Effectively, however a placebo does not contain any pharmacological ingredencies

- What means “randomized"?

For peer review only - http://bmjopen.bmj.com/site/about/guidelines.xhtml

Many studies compare one or several different treatments. For example, one medication is compared with another intervention. Therefore, two groups of participants are created; one group obtains the medication and the other group the intervention. “Randomization” means that it is drawn who comes in which group. With such a test you are allocated by chance to either obtain the medication or the intervention.

- What means “screening"?
  An examination of many people which is conducted in the same manner and in case of this study, it is used to test if women with interest in study participation are indeed suitable for study inclusion and if they fulfill eligibility criteria. The screening will be carried out by surveys.

For peer review only

For peer review only - http://bmjopen.bmj.com/site/about/guidelines.xhtml

Statement of consent

Written statement of consent for participation in the study project
Please read this form thoroughly. Please ask if you do not understand or if you want to know something. For study participation, your written statement of consent is necessary.

**BASEC-number:**	ID 2017-02186
**Title of study**:	**Efficacy study of an integrative and side effect free intervention method of premenstrual complaints**
**Responsible institution**:	**University of Basel**<br>Faculty of Psychology<br>Clinical Psychology and Psychotherapy<br>Missionsstrasse 62a<br>4055 Basel, Switzerland
**Place of conduct:**	**University of Basel**<br>Faculty of Psychology<br>Clinical Psychology and Psychotherapy<br>Missionsstrasse 62a<br>4055 Basel, Switzerland
**Responsible investigator at the study site:**<br>(Antje Frey Nascimento or Cosima Locher)	
**Participant:**<br>Surname and name in block letters:<br><br>Date of birth:	

- I have been informed orally and written by the signing investigator about the purpose, the proceeding of the study, about possible advantages and disadvantages as well as about possible risks.
- My participation in this study is voluntary and I accept the content of the handed out written information. I had enough time to make my decision.
- My questions in association with study participation have been answered. I will keep the written information and obtain a copy of my written consent statement.
- I have been elucidated about the existence of possible other treatments or treatment proceedings.
- I agree that the responsible experts of the competent ethics committee and the intern monitor are allowed to review my uncoded data for audit and control purposes, however only while complying strictly with confidentiality.
- I will be informed about study results which concern my health (e.g., suspicion of the presence of any psychological disorder). If I have any objections in this regard, I will inform the investigator.
- I know that my health-related and personal data can only be forwarded in coded format for research purposes of this study.
- At any time and without disclosure of reasons, I can withdraw from study participation and without the occurrence of any negative consequences. The data which is obtained until withdrawal will be used for data analyses of this study.
- The liability insurance of the Faculty of Psychology will pay in any case of damage.

For peer review only - http://bmjopen.bmj.com/site/about/guidelines.xhtml

- I am aware that the mentioned duties of this participant information have to be met (meeting arranged appointments at study participation, among others). To protect my health the investigator is allowed to exclude me at any time from the study.

Place, date	Signature of participant

Confirmation of the investigator: Herewith I confirm that I explained the essence, meaning and the scope of the study to this participant. I assure to fulfil all duties which are correlated with this study according to the current law. Should I at any times during the conduct of this study find out about any aspects which could influence the willingness of this participant to participate in the study, I will inform her immediately.

Place, date	Surname and name of the investigator in block letters<br><br>Signature of the investigator

For peer review only - http://bmjopen.bmj.com/site/about/guidelines.xhtml

For peer review only

2) STUDY INFORMATION AND INFORMED CONSENT FORM (ORIGINAL; IN GERMAN)

Wirksamkeitsstudie einer integrativen und nebenwirkungsfeien Behandlungsmethode bei prämenstruellen Beschwerden

Diese Studie ist organisiert durch:

Universität Basel
Fakultät für Psychologie
Klinische Psychologie und Psychotherapie
Missionsstrasse 62a
CH-4055 Basel

Sehr geehrte Damen

Wir möchten Sie anfragen, ob Sie an einer klinischen Wirksamkeitsstudie teilnehmen wollen. Im Folgenden wird Ihnen unser Studienvorhaben dargestellt:

18. Ziel der Studie

Wir untersuchen eine neuartige, integrative und nebenwirkungsfeie Behandlungsmethode bei prämenstruellen Beschwerden, die bereits bei Reizdarmsyndrom und chronischen Rückenschmerzen gute Wirksamkeit gezeigt hat. Dabei werden auch Placebodragees eingesetzt.

19. Auswahl

Einschluss:
Es können alle Frauen teilnehmen, die unter einem mittelschweren bis schweren prämenstruellen Syndrom (im folgenden PMS genannt) leiden und zwischen 18 und 45 Jahre alt sind. Sie sollten zudem einen regelmässigen Menstruationszyklus haben. Das PMS sollte so stark sein, dass Sie das Bedürfnis nach einer Behandlung haben und dadurch beeinträchtigt (z.B. durch Arbeitsausfall, Konflikte mit Nahestehenden etc.) sind.

Ausschlusskriterien:
- Sie sind schwanger oder stillen
- Sie leiden unter einer schwerwiegenden psychischen oder körperlichen Erkrankung
- Sie nehmen Drogen oder konsumieren Alkohol in grossen Mengen oder sonstige psychoaktive Substanzen
- Sie haben vor weniger als 30 Tagen mit der Einnahme eines neuen Medikaments begonnen
- Sie sind sterilisiert, Ihnen wurde die Gebärmutter oder die Eierstöcke (teilweise) entfernt
- Sie haben einen Body-Mass-Index von 30 oder höher
- Sie sind wegen Ihren PMS Beschwerden aktuell in psychotherapeutischer Behandlung
- Sie sind allergisch gegen Lactose, Magnesium oder gegen einen der weiteren Inhaltsstoffe der Placebodragees (pharmakologisch unbedenkliche und unwirksame Substanzen)

20. Allgemeine Informationen

Es handelt sich um eine nationale Studie, in welcher eine neue, vielversprechende Behandlungsmethode bei Frauen, welche unter PMS leiden, getestet wird. Dabei werden auch Placebodragees eingesetzt, d.h. Dragees ohne pharmakologischen Wirkstoff. Die Studie besteht aus einer Screeningphase, die ungefähr einen Monat dauert und einer Interventionsphase, die ungefähr zwei Monate dauert. Studienteilnehmerinnen werden täglich für ca. drei Monate ein online Symptomtagebuch ausfüllen (ca. 2 Min./Tag).
Während der Interventionsphase findet ein ambulanter Besuch an der Universität Basel statt (Missionsstrasse 62a, 4055 Basel) welcher ungefähr 60 Minuten dauern wird. Zusätzlich wird es in

For peer review only - http://bmjopen.bmj.com/site/about/guidelines.xhtml

der Interventionsphase zwei telefonische Gespräche und weitere online Befragungen geben. Wir planen 150 Teilnehmerinnen in die Studie einzuschliessen. Bestehende hormonelle oder sonstige beschwerdespezifische Medikation kann fortgeführt werden.

Diese Studie wird so durchgeführt, wie es die Gesetze in der Schweiz vorschreiben. Ausserdem beachten wir alle international anerkannten Richtlinien. Die zuständige Kantonale Ethikkommission hat die Studie bereits geprüft und bewilligt. Eine Beschreibung dieser Studie finden Sie auch auf der Internetseite des Bundesamtes für Gesundheit: www.kofam.ch (*Registrierungsnummer wird hier noch eingefügt nach Bewilligung der Studie).*

21. Ablauf

- Es gibt drei Studiengruppen, von denen Sie einer per Zufall zugeordnet werden: Eine Kontrollgruppe, eine Placebogruppe und eine Behandlungsgruppe. Teilnehmerinnen der Kontrollgruppe haben am Ende der Studie die Möglichkeit, ebenfalls die neuartige Behandlungsmethode zu erhalten.
  Teilnehmerinnern der Placebogruppe erhalten eine Placebobehandlung auf offene Weise (d.h. es wird Ihnen offen gesagt, dass Sie ein Placebo erhalten).
  Teilnehmerinnen der Behandlungsgruppe erhalten eine integrative und nebenwirkungsfreie Behandlungsmethode, bei welcher es ebenfalls zu einer offenen Verabreichung von Placebos kommt.
- Unabhängig von der Studiengruppe werden wir Sie bitten, täglich Fragen eines PMS Tagebuchs online zu beantworten (über insgesamt 3 Monate).
- Zusätzlich werden Sie gebeten, im Rahmen der Screeningphase eine erste online Befragung auszufüllen. Ausserdem werden wir während jeder prämenstruellen Phase weitere online Befragungen durchführen, d.h. über die drei Monate hinweg zu insgesamt drei Zeitpunkten. Zusätzlich wird am Ende der Studie, nach Abschluss der Behandlung, noch eine letzte online Befragung stattfinden.
- Zu Beginn der Interventionsphase wird es einen Besuch bei uns in der Abteilung geben an dem wir, je nach Gruppenzuteilung, die Intervention durchführen. Zuvor führen wir noch ein Screening auf psychische Störungen sowie einen Schwangerschaftstest durch. In der Placebogruppe und Behandlungsgruppe werden dann die Placebodragees ausgehändigt. Nach ca. einem Monat werden wir ein Zwischengespräch mit Ihnen - je nach Wunsch am Telefon oder persönlich - durchführen. Nach dem Abschluss der Studie werden wir Sie noch einmal telefonisch kontaktieren.
- Wenn Sie bereits seit mindestens 30 Tagen Medikamente gegen PMS Beschwerden nehmen, dann dürfen Sie diese, wie gehabt, weiternehmen.
- Die gesamte Studie dauert ca. 3 Monate. Täglich werden sie ungefähr 2 Minuten benötigen um Fragen eines PMS Symptomtagebuchs online auszufüllen. Der Besuch bei uns vor Ort wird ca. 60 Minuten, das Zwischengespräch ca. 15 Minuten und das Endgespräch ca. 10 Minuten dauern. Die weiteren fünf Onlinebefragungen dauern im Schnitt ca. 10 Minuten.
  Tabelle 1 zeigt eine Übersicht der geplanten Kontakte und online Befragungen und die geschätzte Dauer.

Tabelle 1. Studienablauf

**STUDIE**	**Wann?**	**Wo?**	**Inhalt?**	**Wie lange?**
SCREENINGPHASE				
Mündliche/schriftliche Studieninformation	Ganz zu Beginn	Am Telefon/ per Post	Sie werden von uns im Detail über die Studie und Ihre Rechte informiert	Ca. 10 Minuten
Erste Screening Befragung	Direkt nach dem ersten Telefonat	online	Abklärung der Einschlusskriterien	Ca. 20 Minuten

For peer review only - http://bmjopen.bmj.com/site/about/guidelines.xhtml

			Erheben von persönlichen Daten<br><br>Kurzes PMS Screening	
PMS Tagebuch Screening	Während 3 Zyklen	online	Täglich werden Sie gebeten Ihre Symptomintensität und Beeinträchtigungen im Alltag einzuschätzen	Täglich ca. 2 Minuten für mindestens einen Menstruations-zyklus.
PMS Befragung	4. Prämenstruelle Phase	online	Fragebögen	Ca. 10 Minuten
Info über Studienteilnahme	Direkt nach Einsetzen der Menstruation, im zweiten Menstruations-zyklus	Anruf	Studienteilnahme Kriterien erfüllt, Termin für ersten Studienbesuch	Ca. 5 Minuten
INTERVENTIONSPHASE				
Persönlicher Studienbesuch	In den ersten 14 Tagen des zweiten Menstruations-zyklus nach Screening	An der Universität Basel	Letztes Screening von psychischen Störungen und Schwangerschafts-test als Ausschlusskriterium<br><br>Gruppen-spezifische Intervention<br><br>Ausfüllen von Online-Fragebögen nach der Intervention	Ca. 60 Minuten
PMS Befragung	5. Prämenstruelle Phase	online	Fragebögen	Ca. 10 Minuten
Zwischengespräch	Ca. einen Monat nach dem Studienbesuch	Telefon / Skype oder persönlich	Gruppen-spezifische Intervention	Ca. 15 Minuten
PMS Befragung	6. Prämenstruelle Phase	online	Fragebögen	Ca. 10 Minuten
Nachbehandlungs-gespräch	Nach insgesamt ca. drei Monaten	Telefon	Abschluss	Ca. 10 Minuten
Nachbehandlungs-Befragung	Nach dem Nachbehandlungs-gespräch	online	Abschluss-Fragebögen	Ca. 20 Minuten
Zurücksenden der Drageebehälter (ggf. mit übrigen Dragees) in Behandlungsgruppen				
Überweisung der Aufwandsentschädigung				

For peer review only - http://bmjopen.bmj.com/site/about/guidelines.xhtml

22. Nutzen

Mit der Teilnahme an unserer Studie haben Sie die Möglichkeit, an einer neuartigen, integrativen und nebenwirkungsfreien Behandlung teilzunehmen und leisten zudem einen Beitrag zur Erforschung einer nebenwirkungsfreien Behandlungsmethode.
Das alleinige Ausfüllen des Symptomtagebuchs kann bereits helfen, das eigene PMS Symptommanagement zu verbessern.
Zusätzlich können die Resultate wichtig sein für das weitere Verständnis von PMS Interventionen, Wirkfaktoren des therapeutischen Kontexts sowie für die ethische Anwendung von Placebos in der klinischen Forschung.

23. Rechte

Sie nehmen freiwillig teil. Wenn Sie nicht mitmachen oder später Ihre Teilnahme zurückziehen wollen, müssen Sie dies nicht begründen. Bei Abbruch der Studienteilnahme entstehen für Sie keinerlei negative Konsequenzen. Sie dürfen jederzeit Fragen zur Studienteilnahme stellen. Wenden Sie sich dazu bitte an die Personen, die am Ende dieser Information genannt sind.

24. Pflichten

Als Teilnehmerin ist es notwendig, dass Sie sich an die notwendigen Vorgaben und Anforderungen der Studie halten. Dies beinhaltet das Einhalten von vereinbarten Terminen und das zeitnahe Ausfüllen der Onlinebefragungen.
Zudem bitten wir Sie, die Prüfperson über den Verlauf Ihrer Symptome zu informieren und neue Symptome, massive Symptomverschlechterung und sonstige relevante Änderungen im Befinden zu melden. Bitte informieren Sie die Prüfperson über die gleichzeitige Behandlung und Therapie bei Ihrem Arzt und über die Einnahme und Dosisänderung von Medikamenten - auch im Verlauf der Studie.

25. Risiken und Belastungen für die Teilnehmenden

Es sind keine studienspezifischen Risiken zu erwarten. Die Auseinandersetzung mit Ihren Beschwerden könnte möglicherweise zu einer verstärkten Wahrnehmung der Symptome, jedoch auch zu einem besseren Symptommanagement und somit wiederum zur Verbesserung der Beschwerden führen.

Für Frauen, die schwanger werden können

Sollten Sie während der Studie schwanger werden, bitten wir Sie uns direkt zu informieren. Die Studienteilnahme wird dann vorzeitig beendet, da wir Ihre Daten nicht verwenden können.

26. Andere Behandlungsmöglichkeiten

Es existieren erprobte Behandlungsmöglichkeiten für beeinträchtigende prämenstruelle Beschwerden, über die Sie ihr Haus- oder Frauenarzt aufklären kann. Dies ist natürlich auch nach Studienteilnahme möglich.

27. Ergebnisse aus der Studie

Die Prüfperson informiert die Studienteilnehmerinnen während der Studie über alle neuen Erkenntnisse, die den Nutzen dieser Studie und somit die Einwilligung zur Teilnahme beeinflussen können. Bei Zufallsbefunden, die bei Ihnen zur Feststellung und Behandlung bestehender Krankheiten beitragen können, werden Sie informiert. Wenn Sie nicht informiert werden wollen, sprechen Sie bitte vor Studienbeginn mit der Prüfperson.

28. Vertraulichkeit der Daten und Proben

Für diese Studie werden Ihre persönlichen und medizinischen Angaben erfasst.
Bei der Datenerhebung zu Studienzwecken werden die Daten verschlüsselt. Verschlüsselung bedeutet, dass alle Bezugsdaten, die Sie identifizieren könnten (Name, Geburtsdatum), gelöscht und durch einen Schlüssel ersetzt werden. Diejenigen Personen, die den Schlüssel nicht kennen, können daher keine Rückschlüsse auf Ihre Person ziehen. Die Schlüssel-Liste bleibt immer in der Institution (Fakultät für Psychologie). Alle Personen, die Einsicht in Ihre Daten haben, unterliegen

For peer review only - http://bmjopen.bmj.com/site/about/guidelines.xhtml

der Schweigepflicht. Alle Vorgaben des Datenschutzes werden eingehalten und wir werden Ihren Namen weder in einer Publikation noch im Internet öffentlich machen. Sie als teilnehmende Person haben jederzeit das Recht auf Einsicht in Ihre Daten.

Möglicherweise wird diese Studie durch die zuständige Ethikkommission oder durch die Universität Basel überprüft. Die Prüfperson muss eventuell Ihre persönlichen und medizinischen Daten für solche Kontrollen offenlegen.

29. Rücktritt
Sie können jederzeit aufhören und von der Studie zurücktreten, wenn Sie das wünschen. Die bis dahin erhobenen Daten werden noch verschlüsselt ausgewertet, weil das ganze Projekt sonst seinen Wert verliert.
Nach der Auswertung werden Ihre Daten bei Rücktritt vollständig anonymisiert, d.h. Ihre Schlüsselzuordnung wird vernichtet, so dass danach niemand mehr erfahren kann, dass die Daten ursprünglich von Ihnen stammten.

30. Entschädigung für Teilnehmende
Wenn Sie bei dieser Studie mitmachen, bekommen Sie dafür bei vollständiger Teilnahme an der Studie folgende Entschädigung: 200 CHF oder 25 Unterschriften (für Studentinnen der Psychologie). 70 CHF bei der Teilnahme an der gesamten Screeningphase und weitere 130 CHF bei der Teilnahme an der gesamten Interventionsphase inklusive online Befragungen. Auslagen wie Arbeitsausfall oder Reisespesen, die nur durch die Teilnahme bedingt sind, sind in der Entschädigung bereits enthalten. Es entstehen Ihnen oder Ihrer Krankenkasse keine Kosten durch die Teilnahme.

31. Haftung
Die Institution, die die Studie veranlasst hat und für die Durchführung verantwortlich ist, haftet für Schäden, welche Ihnen im Zusammenhang mit der getesteten Substanz oder Forschungshandlungen (z.B. Untersuchungen) entstehen könnten. Die Voraussetzungen und das Vorgehen dazu sind gesetzlich geregelt. Falls Sie einen Schaden erlitten haben, so wenden Sie sich bitte an die Prüfperson.

32. Finanzierung der Studie
Die Studie wird vom Schweizerischen Nationalfonds bezahlt.

33. Kontaktpersonen
Bei Fragen, Unsicherheiten oder Notfällen, die während der Studie oder danach auftreten, können Sie sich jederzeit an eine dieser Kontaktpersonen wenden:

Antje Frey Nascimento M Sc
Missionsstrasse 60/62
Tel: (+41) 061 207 05 98
antje.freynascimento@unibas.ch

Dr. phil. Cosima Locher
Missionsstrasse 60/62
Tel: (+41) 061 207 03 85
cosima.locher@unibas.ch

Studien-E-Mailadresse: pms-studie-psychologie@unibas.ch

34. Glossar von erklärungsbedürftigen Begriffen:
- Was heisst „Placebo"?
  Ein Placebo sieht aus wie ein echtes Medikament und ist auch gleich verpackt. Tatsächlich

For peer review only - http://bmjopen.bmj.com/site/about/guidelines.xhtml

enthält ein Placebo aber keine pharmakologischen Wirkstoffe.

- Was heisst „randomisiert"?
Bei vielen Studien werden zwei oder mehrere unterschiedliche Arten der Behandlung verglichen. Zum Beispiel vergleicht man ein Medikament mit einer anderen Intervention. Man bildet dann zwei Gruppen von Teilnehmenden, die einen bekommen das Medikament und die anderen die andere Intervention. „Randomisieren" bedeutet dann, dass ausgelost wird, wer in welche Gruppe kommt. Es ist bei einem solchen Test also Zufall, ob man das Medikament erhält oder die andere Intervention.

- Was heisst „Screening"?
Eine Untersuchung, die an vielen Personen in der gleichen Weise durchgeführt wird und im Fall dieser Studie, um zu prüfen, ob Frauen mit Interesse an der Studienteilnahme auch wirklich für diese Studie geeignet sind und die Einschlusskriterien erfüllen. Das Screening wird durch Befragungen durchgeführt.

For peer review only

For peer review only - http://bmjopen.bmj.com/site/about/guidelines.xhtml

Einwilligungserklärung

Schriftliche Einwilligungserklärung zur Teilnahme an einem Studienprojekt
Bitte lesen Sie dieses Formular sorgfältig durch. Bitte fragen Sie, wenn Sie etwas nicht verstehen oder wissen möchten. Für die Teilnahme ist Ihre schriftliche Einwilligung notwendig.

**BASEC-Nummer:**	ID 2017-02186
**Titel der Studie**:	**Wirksamkeitsstudie einer integrativen und nebenwirkungsfeien Behandlungsmethode bei prämenstruellen Beschwerden**
**verantwortliche Institution**:	**Universität Basel**<br>Fakultät für Psychologie<br>Klinische Psychologie und Psychotherapie<br>Missionsstrasse 62a<br>CH-4055 Basel
**Ort der Durchführung**:	**Universität Basel**<br>Fakultät für Psychologie<br>Klinische Psychologie und Psychotherapie<br>Missionsstrasse 62a<br>CH-4055 Basel
**verantwortliche Prüfperson am Studienort:**<br>(Antje Frey Nascimento oder Cosima Locher)	
**Teilnehmerin:**<br>Name und Vorname in Druckbuchstaben:<br><br>Geburtsdatum:	

- Ich wurde von der unterzeichnenden Prüfperson mündlich und schriftlich über den Zweck, den Ablauf der Studie, über mögliche Vor- und Nachteile sowie über eventuelle Risiken informiert.
- Ich nehme an dieser Studie freiwillig teil und akzeptiere den Inhalt der abgegebenen schriftlichen Information. Ich hatte genügend Zeit, meine Entscheidung zu treffen.
- Meine Fragen im Zusammenhang mit der Teilnahme an dieser Studie sind mir beantwortet worden. Ich behalte die schriftliche Information und erhalte eine Kopie meiner schriftlichen Einwilligungserklärung.
- Ich wurde über die Existenz möglicher anderer Behandlungen und Behandlungsverfahren aufgeklärt.
- Ich bin einverstanden, dass die zuständigen Fachleute der zuständigen Ethikkommission und der interne Monitor zu Prüf- und Kontrollzwecken in meine unverschlüsselten Daten Einsicht nehmen dürfen, jedoch unter strikter Einhaltung der Vertraulichkeit.
- Bei Studienergebnissen, die direkt meine Gesundheit betreffen (z.B. Verdacht auf Vorliegen einer psychischen Erkrankung), werde ich informiert. Wenn ich das nicht wünsche, informiere ich meine Prüfperson.
- Ich weiss, dass meine gesundheitsbezogenen und persönlichen Daten nur in verschlüsselter Form zu Forschungszwecken für diese Studie weitergegeben werden können.
- Ich kann jederzeit und ohne Angabe von Gründen von der Studienteilnahme zurücktreten und ohne dass daraus negative Konsequenzen für mich entstehen. Die bis zum Rücktritt erhobenen Daten werden für die Auswertung zur Studie verwendet.
- Die Haftpflichtversicherung der Fakultät für Psychologie kommt für allfällige Schäden auf.

For peer review only - http://bmjopen.bmj.com/site/about/guidelines.xhtml

- Ich bin mir bewusst, dass die in der Teilnehmerinformation genannten Pflichten einzuhalten sind (u.a. Einhalten vereinbarter Termine bei Studienteilnahme). Im Interesse meiner Gesundheit kann mich die Prüfperson jederzeit von der Studie ausschliessen.

Ort, Datum	Unterschrift Teilnehmerin

Bestätigung der Prüfperson: Hiermit bestätige ich, dass ich dieser Teilnehmerin Wesen, Bedeutung und Tragweite der Studie erläutert habe. Ich versichere, alle im Zusammenhang mit dieser Studie stehenden Verpflichtungen gemäss des geltenden Rechts zu erfüllen. Sollte ich zu irgendeinem Zeitpunkt während der Durchführung der Studie von Aspekten erfahren, welche die Bereitschaft der Teilnehmerin zur Teilnahme an der Studie beeinflussen könnten, werde ich sie umgehend darüber informieren.

Ort, Datum	Name und Vorname der Prüfperson in Druckbuchstaben<br><br>Unterschrift der Prüfperson

For peer review only - http://bmjopen.bmj.com/site/about/guidelines.xhtml

3) ORAL INFORMATION OF THE OPEN-LABEL PLACEBO TREATMENT RATIONALE

The following is told to participants of the open-label placebo with treatment rationale study group at the study visit, after participants are informed about their group allocation:

I. **Placebos can be powerful**

"Our treatment follows a completely new concept: the open administration of placebos.

In the following, I want to explain to you why we conduct this study. We know already that the placebo effect can be high if drugs are tested against placebos. Scientist used to believe that placebo pills only work if you administer placebos deceptively, hence, with deception. Now new evidence suggests that this is not true. We are currently testing this in regard of the premenstrual syndrome (PMS).

a. First, a large body of studies on double-blind randomized trials shows that the placebo effect is powerful – for many complaints! This means that placebos can alleviate, among others, symptoms such as pain, cramps, intestinal complaints, and also may have a very positive effect on mood.
b. Substantial placebo effects also could be found for PMS and even for a severe subtype of PMS – for the premenstrual dysphoric syndrome. Many PMS intervention studies reveal that about 40 % of the treatment effect can be led back merely to placebo effects. In addition, in single studies placebos have been even more effective than the pharmacological treatment. In those studies, neither the patient nor the investigator had known whether patients obtained the tested medication or placebos. We are now trying open-label placebos for the first time in PMS and we think that the treatment can be effective.

II. **Why placebos work**

Next, I would like to explain to you why placebos can alleviate complaints. A very important explanation is that the body is able to react automatically to the intake of medication. Very early in life we learn already that pills and an alleviating effect are associated. Finally, we achieve already a pain-relieving effect just by swallowing pills. Comparable is the physiological reaction of our body after placebo intake. We know that when placebo pills work they activate neurotransmitters like

For peer review only - http://bmjopen.bmj.com/site/about/guidelines.xhtml

endorphins, dopamine, and endocannabinoids and specific regions of the brain automatically. In turn, these neurotransmitters and brain regions are capable of alleviating pain or of affecting mood.

III. Importance of faithful intake

It is absolutely ok if you bear any doubts that the placebos will work. Hence, if placebos work it will happen automatically, also if you have any doubts. However, it is crucial to take the placebos faithfully to obtain an effect, i.e., regularly and as prescribed. This means for you that you should take the placebo pills now faithfully to feel any effect. Also, it is important to be aware about the fact that some experience an alleviation earlier and others only later. Therefore, the regular intake is very important. If you take the pills we also recommend to remember for what exactly the pills should help you.

IV. Open-label placebos

The most of what we know about placebos we know from research of deceptive placebo administration. Yet, several studies show that placebos even work if you know that you take placebos. We are adopting this approach in our study. Good efficacy for an open placebo intervention were already found especially for irritable bowel syndrome (IBS) and chronic low back pain. Even in patients who did not respond to usual treatment. In particular, in such cases it can be assumed that self-healing processes can be activated due to the placebo intervention.

For our study, which is funded by the *Swiss National Science Foundation*, we work together with the placebo research group from *Harvard Medical School*, which is leading in this innovative intervention approach.

Now, I would like to show you a video sequence from the American *NBC News*. In this news sequence the work of the placebo research group from *Harvard Medical School* is presented and their work of an open placebo intervention for IBS.

[*short video sequence is shown with German subtitle]*

In the video sequence you could see that patients with IBS obtained 2x2 pills for three weeks. Since PMS complaints have a cyclical nature, you will receive pills for six weeks and you will be asked to take two pills a day – this is closer to the common pharmacological treatments of PMS.

For peer review only - http://bmjopen.bmj.com/site/about/guidelines.xhtml

I am aware that it may sound strange at first place that you should take placebos. However, we want to find out what will happen to your complaints if you take placebo pills on a daily basis. Therefore, I would like to encourage you to give a chance to the open placebo intervention and see what will happen."

[*further intake instructions are given]*

For peer review only

For peer review only - http://bmjopen.bmj.com/site/about/guidelines.xhtml

Reporting checklist for protocol of a clinical trial.

Based on the SPIRIT guidelines.

Instructions to authors

Complete this checklist by entering the page numbers from your manuscript where readers will find each of the items listed below.

Your article may not currently address all the items on the checklist. Please modify your text to include the missing information. If you are certain that an item does not apply, please write "n/a" and provide a short explanation.

Upload your completed checklist as an extra file when you submit to a journal.

In your methods section, say that you used the SPIRITreporting guidelines, and cite them as:

Chan A-W, Tetzlaff JM, Altman DG, Laupacis A, Gøtzsche PC, Krleža-Jerić K, Hróbjartsson A, Mann H, Dickersin K, Berlin J, Doré C, Parulekar W, Summerskill W, Groves T, Schulz K, Sox H, Rockhold FW, Rennie D, Moher D. SPIRIT 2013 Statement: Defining standard protocol items for clinical trials. Ann Intern Med. 2013;158(3):200-207

		Reporting Item	Page Number
**Administrative information**			
Title	#1	Descriptive title identifying the study design, population, interventions, and, if applicable, trial acronym	1
Trial registration	#2a	Trial identifier and registry name. If not yet registered, name of intended registry	2
Trial registration: data set	#2b	All items from the World Health Organization Trial Registration Data Set	2
Protocol version	#3	Date and version identifier	2
Funding	#4	Sources and types of financial, material, and other support	26
Roles and responsibilities: contributorship	#5a	Names, affiliations, and roles of protocol contributors	1; 26

For peer review only

Roles and responsibilities: sponsor contact information	#5b	Name and contact information for the trial sponsor	n/a
Roles and responsibilities: sponsor and funder	#5c	Role of study sponsor and funders, if any, in study design; collection, management, analysis, and interpretation of data; writing of the report; and the decision to submit the report for publication, including whether they will have ultimate authority over any of these activities	26
Roles and responsibilities: committees	#5d	Composition, roles, and responsibilities of the coordinating centre, steering committee, endpoint adjudication committee, data management team, and other individuals or groups overseeing the trial, if applicable (see Item 21a for data monitoring committee)	26
**Introduction**			
Background and rationale	#6a	Description of research question and justification for undertaking the trial, including summary of relevant studies (published and unpublished) examining benefits and harms for each intervention	5
Background and rationale: choice of comparators	#6b	Explanation for choice of comparators	8; 12
Objectives	#7	Specific objectives or hypotheses	7
Trial design	#8	Description of trial design including type of trial (eg, parallel group, crossover, factorial, single group), allocation ratio, and framework (eg, superiority, equivalence, non-inferiority, exploratory)	9
**Methods: Participants, interventions, and outcomes**			
Study setting	#9	Description of study settings (eg, community clinic, academic hospital) and list of countries where data will be collected. Reference to where list of study sites can be obtained	9
Eligibility criteria	#10	Inclusion and exclusion criteria for participants. If applicable, eligibility criteria for study centres and individuals who will	9

For peer review only

		perform the interventions (eg, surgeons, psychotherapists)	
Interventions: description	#11a	Interventions for each group with sufficient detail to allow replication, including how and when they will be administered	11
Interventions: modifications	#11b	Criteria for discontinuing or modifying allocated interventions for a given trial participant (eg, drug dose change in response to harms, participant request, or improving / worsening disease)	n/a
Interventions: adherance	#11c	Strategies to improve adherence to intervention protocols, and any procedures for monitoring adherence (eg, drug tablet return; laboratory tests)	18; 23
Interventions: concomitant care	#11d	Relevant concomitant care and interventions that are permitted or prohibited during the trial	12
Outcomes	#12	Primary, secondary, and other outcomes, including the specific measurement variable (eg, systolic blood pressure), analysis metric (eg, change from baseline, final value, time to event), method of aggregation (eg, median, proportion), and time point for each outcome. Explanation of the clinical relevance of chosen efficacy and harm outcomes is strongly recommended	15 ff.
Participant timeline	#13	Time schedule of enrolment, interventions (including any run-ins and washouts), assessments, and visits for participants. A schematic diagram is highly recommended (see Figure)	11
Sample size	#14	Estimated number of participants needed to achieve study objectives and how it was determined, including clinical and statistical assumptions supporting any sample size calculations	21
Recruitment	#15	Strategies for achieving adequate participant enrolment to reach target sample size	10
**Methods: Assignment of interventions (for controlled trials)**			
Allocation: sequence generation	#16a	Method of generating the allocation sequence (eg, computer-generated random numbers), and list of any factors for stratification. To reduce predictability of a random sequence, details of any planned restriction (eg, blocking) should be provided in a separate document that is unavailable to those who enrol participants or assign interventions	11

For peer review only

Allocation concealment mechanism	#16b	Mechanism of implementing the allocation sequence (eg, central telephone; sequentially numbered, opaque, sealed envelopes), describing any steps to conceal the sequence until interventions are assigned	12
Allocation: implementation	#16c	Who will generate the allocation sequence, who will enrol participants, and who will assign participants to interventions	12
Blinding (masking)	#17a	Who will be blinded after assignment to interventions (eg, trial participants, care providers, outcome assessors, data analysts), and how	n/a
Blinding (masking): emergency unblinding	#17b	If blinded, circumstances under which unblinding is permissible, and procedure for revealing a participant's allocated intervention during the trial	n/a
**Methods: Data collection, management, and analysis**			
Data collection plan	#18a	Plans for assessment and collection of outcome, baseline, and other trial data, including any related processes to promote data quality (eg, duplicate measurements, training of assessors) and a description of study instruments (eg, questionnaires, laboratory tests) along with their reliability and validity, if known. Reference to where data collection forms can be found, if not in the protocol	13 ff.
Data collection plan: retention	#18b	Plans to promote participant retention and complete follow-up, including list of any outcome data to be collected for participants who discontinue or deviate from intervention protocols	n/a
Data management	#19	Plans for data entry, coding, security, and storage, including any related processes to promote data quality (eg, double data entry; range checks for data values). Reference to where details of data management procedures can be found, if not in the protocol	21
Statistics: outcomes	#20a	Statistical methods for analysing primary and secondary outcomes. Reference to where other details of the statistical analysis plan can be found, if not in the protocol	22
Statistics: additional analyses	#20b	Methods for any additional analyses (eg, subgroup and adjusted analyses)	22

For peer review only

Statistics: analysis population and missing data	#20c	Definition of analysis population relating to protocol non-adherence (eg, as randomised analysis), and any statistical methods to handle missing data (eg, multiple imputation)	22
**Methods: Monitoring**			
Data monitoring: formal committee	#21a	Composition of data monitoring committee (DMC); summary of its role and reporting structure; statement of whether it is independent from the sponsor and competing interests; and reference to where further details about its charter can be found, if not in the protocol. Alternatively, an explanation of why a DMC is not needed	23
Data monitoring: interim analysis	#21b	Description of any interim analyses and stopping guidelines, including who will have access to these interim results and make the final decision to terminate the trial	n/a
Harms	#22	Plans for collecting, assessing, reporting, and managing solicited and spontaneously reported adverse events and other unintended effects of trial interventions or trial conduct	20
Auditing	#23	Frequency and procedures for auditing trial conduct, if any, and whether the process will be independent from investigators and the sponsor	23
**Ethics and dissemination**			
Research ethics approval	#24	Plans for seeking research ethics committee / institutional review board (REC / IRB) approval	26
Protocol amendments	#25	Plans for communicating important protocol modifications (eg, changes to eligibility criteria, outcomes, analyses) to relevant parties (eg, investigators, REC / IRBs, trial participants, trial registries, journals, regulators)	n/a
Consent or assent	#26a	Who will obtain informed consent or assent from potential trial participants or authorised surrogates, and how (see Item 32)	23
Consent or assent: ancillary studies	#26b	Additional consent provisions for collection and use of participant data and biological specimens in ancillary studies, if applicable	n/a
Confidentiality	#27	How personal information about potential and enrolled participants will be collected, shared, and maintained in order to protect confidentiality before, during, and after the trial	23; 21

For peer review only

Declaration of interests	#28	Financial and other competing interests for principal investigators for the overall trial and each study site	26
Data access	#29	Statement of who will have access to the final trial dataset, and disclosure of contractual agreements that limit such access for investigators	n/a
Ancillary and post trial care	#30	Provisions, if any, for ancillary and post-trial care, and for compensation to those who suffer harm from trial participation	n/a
Dissemination policy: trial results	#31a	Plans for investigators and sponsor to communicate trial results to participants, healthcare professionals, the public, and other relevant groups (eg, via publication, reporting in results databases, or other data sharing arrangements), including any publication restrictions	2
Dissemination policy: authorship	#31b	Authorship eligibility guidelines and any intended use of professional writers	n/a
Dissemination policy: reproducible research	#31c	Plans, if any, for granting public access to the full protocol, participant-level dataset, and statistical code	n/a
**Appendices**			
Informed consent materials	#32	Model consent form and other related documentation given to participants and authorised surrogates	n/a
Biological specimens	#33	Plans for collection, laboratory evaluation, and storage of biological specimens for genetic or molecular analysis in the current trial and for future use in ancillary studies, if applicable	n/a

The SPIRIT checklist is distributed under the terms of the Creative Commons Attribution License CC-BY-ND 3.0. This checklist was completed on 09. July 2019 using https://www.goodreports.org/, a tool made by the EQUATOR Network in collaboration with Penelope.ai

For peer review only
